# Transcriptome profiling of litchi leaves in response to low temperature reveals candidate regulatory genes and key metabolic events during floral induction

**DOI:** 10.1186/s12864-017-3747-x

**Published:** 2017-05-10

**Authors:** Hongna Zhang, Jiyuan Shen, Yongzan Wei, Houbin Chen

**Affiliations:** 1 College of Horticulture, South China Agricultural University, Guangzhou, 510642 China; 2 Key Laboratory of Tropical Fruit Biology (Ministry of Agriculture), South Subtropical Crops Research Institute, Chinese Academy of Tropical Agricultural Sciences, Zhanjiang, 524091 China

**Keywords:** Litchi (*Litchi chinensis* Sonn.), Floral induction, Transcriptome, Differentially expressed gene (DEG), Low temperature, Leaf

## Abstract

**Background:**

Litchi (*Litchi chinensis* Sonn.) is an economically important evergreen fruit tree widely cultivated in subtropical areas. Low temperature is absolutely required for floral induction of litchi, but its molecular mechanism is not fully understood. Leaves of litchi played a key role during floral induction and could be the site of low temperature perception. Therefore, leaves were treated under different temperature (15 °C/25 °C), and high-throughput RNA sequencing (RNA-Seq) performed with leaf samples for the *de novo* assembly and digital gene expression (DGE) profiling analyses to investigate low temperature-induced gene expression changes.

**Results:**

83,107 RNA-Seq unigenes were *de novo* assembled with a mean length of 1221 bp and approximately 61% of these unigenes (50,345) were annotated against public protein databases. Differentially-expressed genes (DEGs) under low temperature treatment in comparison with the control group were the main focus of our study. Hierarchical clustering analysis arranged 2755 DEGs into eight groups with three significant expression clusters (*p*-value ≤ 0.05) during floral induction. With the increasing contents of sugars and starch, the expression of genes involved in metabolism of sugars increased dramatically after low temperature induction. One *FT* gene (*Unigene0025396*, *LcFT1*) which produces a protein called ‘florigen’ was also detected among DEGs of litchi. *LcFT1* exhibited an apparent specific tissue and its expression was highly increased after low temperature induction, GUS staining results also showed GUS activity driven by *LcFT1* gene promoter can be induced by low temperature, which indicated *LcFT1* probably played a pivotal role in the floral induction of litchi under low temperature.

**Conclusions:**

Our study provides a global survey of transcriptomes to better understand the molecular mechanisms underlying changes of leaves in response to low temperature induction in litchi. The analyses of transcriptome profiles and physiological indicators will help us study the complicated metabolism of floral induction in the subtropic evergreen plants.

**Electronic supplementary material:**

The online version of this article (doi:10.1186/s12864-017-3747-x) contains supplementary material, which is available to authorized users.

## Background

Litchi (*Litchi chinensis Sonn*.), an evergreen fruit tree, is widely distributed in the subtropics, including South China, South Africa and Australia. Floral induction of litchi occurs from late autumn to early winter. After exposure to low temperature for several weeks to induce flowering, litchi trees start floral initiation and morphological development with ascending temperature, subsequently, bloom in spring with favorable conditions [1]. Previous studies have shown that low temperature is a crucial environmental cue for floral induction of subtropical evergreen plants, litchi [2–6], olive [7], mango [8, 9], citrus [10–12] and orchid [13], all of which require a certain period of relatively low temperature to induce flowering. However, besides low temperature, internal factors like state of branches (including buds and leaves) can also result in irregularities in litchi flowering [14]. Most studies on flowering focus on bud because it is the site of flower formation [15–17]. An earlier research involved flowering transition of the buds in litchi had been studied by us [18]. But for litchi, what’s more interesting is the induction effect of flowering under low temperature may be affected by the status of leaves. Recent advances indicate that FT protein (“florigen”) is synthesized in the leaf vasculature, and moves through the phloem to the shoot apical meristem [19–21]. The role of leaves in sensing photoperiod to induce flower has been established in model plants [21–23]. So far, there are still plenty of unresolved questions about litchi flowering, knowledge of the molecular events associated with the low temperature signals that activate flowering of evergreen plants remains obscure, and the events occurring in leaves of litchi during floral induction should be worth exploring.

In recent years, RNA-Seq technology has been widely used to acquire a global view of transcriptomic dynamics for non-model organisms for which there is no reference genome [24–26]. *De novo* assembly and DGE profiling analyses from RNA-Seq data have enabled researchers to more easily obtain information about the expression level between samples or during a particular process [27–29]. This method has been successfully applied to identify genes related to color development in Chinese bayberry (*Myrica rubra*) [30], fruit development and maturation in date palm (*Phoenix dactylifera*, L.) [31] and Chinese white pear (*Pyrus bretschneideri* Rehd) [32], bud dormancy analysis in Japanese pear (*Pyrus pyrifolia* Nakai) and ‘Suli’ pear (*Pyrus pyrifolia* white pear group) [27, 29], fruitlet abscission in litchi (*Litchi chinensis*) and melon (*Cucumis melo*) [33, 34], and floral transition in litchi (*Litchi chinensis*) and bamboo (*Dendrocalamus latiflorus*) [16, 18]. Though litchi fruit and flower transcriptome sequencing data have been published [18, 33], the events in leaves are still unknown, despite the fact that leaves play important roles in sensing low temperature during floral induction. To better understand the molecular mechanisms involved in the litchi floral induction process, we used RNA-Seq to identify the expression of a large number of genes, especially those differentially expressed under low temperature induction.

In the present study, we constructed five cDNA libraries from litchi leaves that had been subjected to different temperatures. Global gene expression profiles during floral induction of litchi were analyzed by RNA-Seq, and the molecular events occurring in leaves related to temperature-dependent floral induction were characterized.

## Methods

### Plant materials

**Fig. 1:**
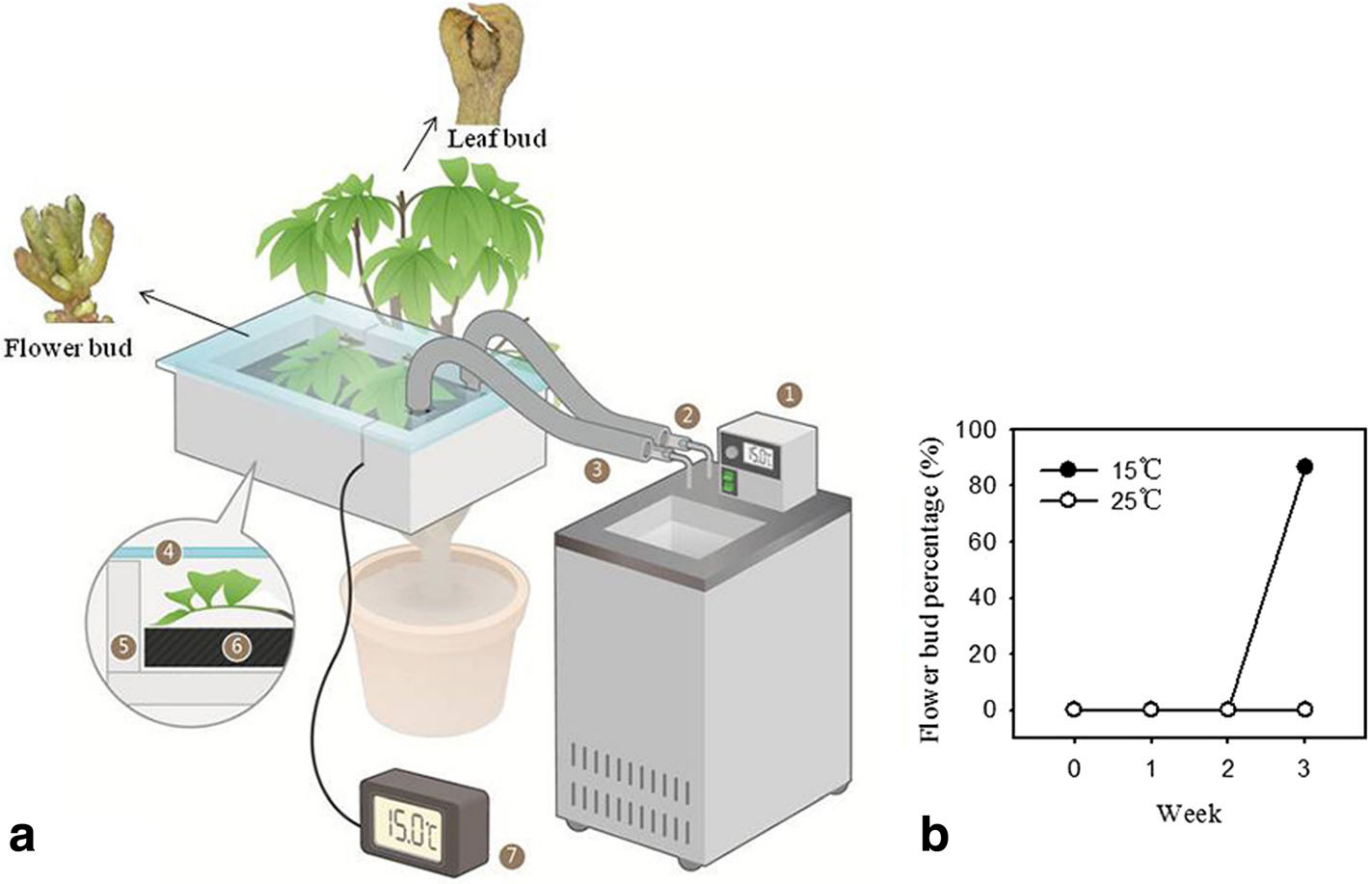
Schematic diagram and flower bud percentage of ‘Feizixiao’ litchi under different temperatures. **a** Schematic diagram of working principle and structural element of the mini temperature control box. The mini temperature control box includes thermostat water bath with function of refrigeration cycle①, plastic tube②, thermal insulation material③, transparency glass plate④, thermal containers⑤, radiators⑥ and electronic thermometer with the probe⑦. The collected images were authentic and original from the truest experiment, by using a drawing tool Coreldraw x4. **b** Percentage of flower buds in ‘Feizixiao’ litchi at different time points at 15 °C and 25 °C

Potted plants of ‘Feizixiao’ litchi produced from air layers were grown in a plastic room with the temperature set at 25 °C under natural sunlight. Two year old plants with mature autumnal terminal shoots were selected for treatments in October 2013. Leaves for the low temperature treatment were enclosed in a mini temperature controlled box where the temperature was set at 15 °C. A schematic diagram of the design and structure of the mini temperature-controlling box is shown in Fig. 1a. The mini box has a thermostatically controlled refrigerated water bath and so can maintain temperature control. To eliminate light influence, the head cover of the mini box was designed with transparent glass plate. To eliminate humidity influence, the humidity of the room was set with that of the mini box. Leaves from the same branches but outside of the box (25 °C) were taken as controls. The temperatures inside (15 °C) and outside (25 °C) of the boxes were maintained over 1 month. The leaves were sampled at 0, 2 and 3 weeks and named 0W, 2W + V, 3W + V, 2W-V and 3W-V, with + V and -V denoting the temperature of 15 °C and 25 °C respectively. The apical buds swelled and attained the floral initiation state at the third week after treatment (Fig. 1a). Unlike *Lotus japonicas* and *Arabidopsis* [35, 36], our previous studies have showed that the expression of *Flowering Locus T* in litchi is not regulated by the circadian clock (Additional file 1), so a total of 10 leaves from each treatment were taken at a regular time on each sampling date. All samples were frozen in liquid nitrogen and stored at -80 °C until further use.

### RNA extraction, library construction and RNA-Seq

RNA was extracted from each sample of three biological replicates using the Quick RNA Isolation Kit (Huayueyang, China) according to the manufacturer’s instructions. Total RNA from five samples was pooled as described in Additional file 2 for library construction, and sequencing was performed by staff at the Beijing Genome Institute using Illumina HiSeq™ 2000 (San Diego, CA, USA). Each pool was sequenced once technically in view of the high repeatability and relatively little technical variation of RNA-Seq data [27, 37].

### Bioinformatics analysis

Raw reads obtained by the HiSeq 2000 were filtered to exclude low complexity reads. The total of clean reads from five libraries were pooled and subjected to *de novo* assembly using Trinity [38] and the TGICL [39]. The *de novo* assembly workflow is presented in Additional file 2. The assembled unigenes were annotated by BLASTx (*E*-value < 10^−5^) against the NCBI non-redundant (Nr) database, Gene ontology (GO), the Swiss-Prot protein database (Swiss-Prot), Clusters of Orthologous Groups (COG) and Kyoto Encyclopedia of Genes and Genomes (KEGG) [40]. The unigene expression was normalized by calculating the read per kilobase per million (RPKM) [41]. After the RPKM of each gene was calculated, differential expression analysis was conducted using edgeR [42]. The threshold of differential expression of unigenes between the samples was set as FDR < 0.001,|log_2_| ≥ 1, and *P*-value < 0.01. The all DEGs between stages were further subjected to GO enrichment analysis and KEGG pathway enrichment analysis to verify biological significance. Functional annotations of unigenes by GO were carried out using the Blast2GO software [43] and WEGO software [44], then all unigenes were mapped to terms in the KEGG database by Blastall software [45]. The *P*-value ≤ 0.05/Q value ≤ 0.05 was used as the threshold to determine significant GO/KEGG enrichment of the gene sets. Gene expression trends from 0W to 3W + V were analyzed and clustered using the software of Short Time-series Expression Miner (STEM) [46]. Then profiles with *P* <0.05 were separately subjected to KEGG pathway enrichment, and top five most significant pathways were focused further [47]. In addition, the transcriptomic data supporting the results of this article are available in the NCBI Sequence Read Archive (SRA) under the accession number SRR2960334 (http://www.ncbi.nlm.nih.gov/sra/). The assembled unigene sequences have been deposited at Transcriptome Shotgun Assembly (TSA) under the accession number GFMD00000000.1 (https://www.ncbi.nlm.nih.gov/genbank/).

### Analysis of sugars

Determination of sugar and starch content was performed according to previous methods [48–50]. 0.3 g samples were homogenized in 90% (v/v) ethanol at 80 °C for 20 min, and centrifuged at 4000 rpm for 10 min. The supernatants were collected, combined, filtered on a Sep-Pak^®^1cc(100mg) C18 Cartridge to remove pigments and other nonsugar compounds, and finally analyzed by an Agilent 1200 HPLC system (Agilent Technologies, Waldbronn, Germany) [48, 49]. Starch was extracted from the residue of sugars with 80% Ca (NO_3_)_2_, stained using the iodine-starch method, and the concentration was evaluated by measuring the absorbance at 620 nm [50].

### RT-qPCR analysis

The transcriptomic data was confirmed using RT-qPCR analysis of 13 selected DEGs during floral induction in litchi. Gene-specific primers were designed using the Primer 5.0 program (Additional file 3). cDNA was synthesized from total RNA (2 μg) using SuperScript® IV Reverse Transcriptase Kit (Thermo Fisher Scientific, USA) following the manufacturer’s instructions. Unit reaction conditions: 50 mM Tris-HCl (pH 8.3), 4 mM MgCl2, 10 mM DTT, 50 mM KCl, 0.5 mM dTTP, 0.4 MBq/mL [3H]-dTTP, 0.4 mM poly(A) oligo (dT)15 and enzyme in 20 μl for 10 min at 37 °C. The RT-qPCR reactions were performed on a LightCycler 1.5 instrument (Roche, USA) using the following program: hot start at 95 °C for 7 min, followed by 40 cycles of 10 s at 95 °C, 55 °C for 15 s, and 25 s at 72 °C. The unigene expression levels were calculated by using the 2^-ΔΔCT^ method [51] and normalized to the actin gene [HQ615689] [52]. Values for each time point was the average of three technical replicates of each biological replicate. Statistical and correlation analysis was performed with SPSS 16.0.

### Promoter analysis of LcFT1 and histochemical GUS assay

Based on the litchi genome databases established by our teams, the promoter sequences of *LcFT1* were obtained. Promoter analysis of cis-acting elements was carried out with online software Plant CARE. The promoter fragment was inserted into a pBI101-GUS vector with the In-Fusion ®HD Cloning Kit (Takara, Japan), resulting in the LcFT1 pro::GUS vector. LcFT1 pro::GUS was transferred into A. tumefaciens EHA105 by freeze-thaw method, and then used to transform Arabidopsis by the floral dip method [53]. GUS staining of whole developing seedlings of the T2 transgenic Arabidopsis plants and Columbia plants with low temperature-treated was performed as described previously [54], and the samples were examined by stereomicroscopy (Leica M80).

## Results

### Temperature-dependent floral induction of ‘Feizixiao’ litchi

The status of apical buds was observed in order to identify DEGs during floral induction in litchi. No flower buds were observed on apical shoots until 3 weeks after low temperature treatment (Fig. 1a). At that time, more than 86.6% of the flower buds had broken on apical shoots under 15 °C treatment (Fig. 1b), whereas no flower buds were found on control shoots.

### Sample preparation and cDNA library construction

To obtain an overview of the litchi leaf transcriptome during floral induction, five libraries (0W, 2W + V, 3W + V, 2W-V and 3W-V) were prepared for RNA-Seq. An overview of the sequencing and assembly is outlined in Additional file 4. A total of 103,924,174 pooled reads from five libraries were subjected to *de novo* assembly. The quality of the reads was assessed using the base-calling quality scores from the Illumina’s base-caller Bustard [33]. In addition, 99.14% of the clean reads had Phred-like quality scores at the Q20 level. The consensus assembly from the five samples generated a total of 97,449 contigs with average length of 941 bp, which were assembled into 83,107 unigenes using paired-end joining and gap-filling, with an N50 of 1552 bp (Additional file 4). The size distribution of these contigs and unigenes is shown in Additional file 5. These data showed that the throughput and sequencing quality was sufficient for further analysis.

### Annotation of predicted proteins

**Fig. 2:**
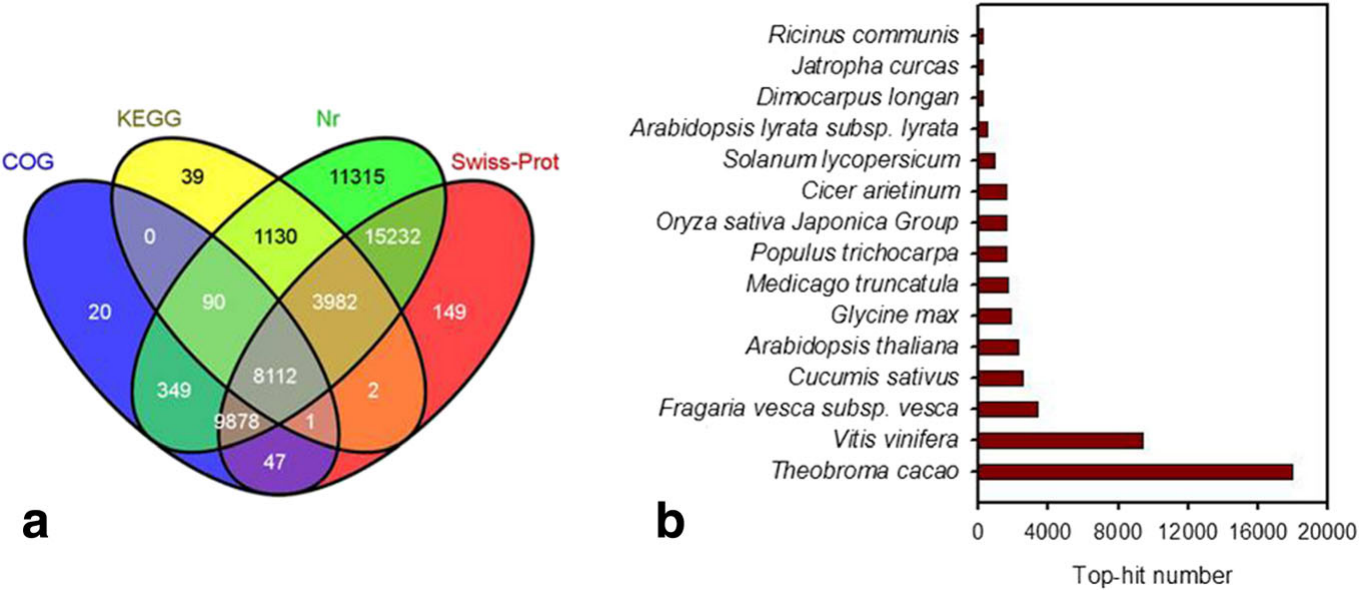
Characteristics of homology search of litchi unigenes. **a** Venn diagram of number of unigenes annotated by BLASTx against protein databases with an *E*-value threshold of 10^-5^. The numbers in the circles indicate the number of unigenes annotated by single or multiple databases. **b** *E*-value distribution of the top BLASTx hits for each unique sequence

Approximately 60% of unigenes (50,345) were annotated based on BLASTx (*E*-value < 10^-5^) searches from four public databases. 50,088 (99.4%) unigenes could be annotated with reference to the Nr database, while 8112 (16.1%) unigenes could be annotated using all the databases (Fig. 2a). The 15 top-hit species based on Nr annotation are shown in Fig. 2b. Nearly 67.0% of unigenes could be annotated to sequences from four top-hit species, i.e., *Theobroma cacao*, *Vitis vinifera*, *Fragaria vesca* and *Cucumis sativus*. 17,991 unigenes (36.0%) had top matches to sequences from *Theobroma cacao*.

### Functional classification

We used GO, COG and KEGG to classify the functions of the predicted litchi unigenes. 21,748 unigenes were classified into three main categories: biological process, cellular component, and molecular function (Additional file 6). In the biological process category, ‘cellular process’, ‘metabolic process’ and ‘response to stimulus’ represented the major categories. For the cellular component category, large numbers of unigenes were categorized as ‘cell’, ‘cell part’ and ‘organelle’. Under the molecular function category, many higher abundant unigenes were classified into ‘binding’ and ‘catalytic activity’ subcategories. The COGs database was used to predict and classify possible functions of proteins. Overall, 18,497 unigenes were classified into 25 COG categories. As shown in Additional file 7, group K (Transcription factors), group L (Replication recombination and repair), and group T (Signal transduction mechanisms) were the three most abundant groups.

KEGG pathway-based analysis was used to further understand the biological functions of genes. We mapped the annotated sequences to the reference standard pathways, and found 13,356 unigenes predicted in 272 pathways, among which ‘RNA transport’ represented the largest pathway (434, 3.25%), followed by ‘Ribosome’ (348, 2.61%), and ‘Starch and sucrose metabolism’ (344, 2.58%). In addition to the ‘Starch and sucrose metabolism’ pathway, many pathways involved in carbohydrate metabolism were also found, such as ‘Glycolysis/gluconeogenesis’ (K000010), ‘Amino sugar and nucleotide sugar metabolism’ (K000520), ‘Fructose and mannose metabolism’ (K000051), ‘Galactose metabolism’ (K000052) and ‘Pentose phosphate pathway’ (K000030) (Additional file 8). Further research into understanding metabolic pathways in this species can be based on the KEGG pathway annotation.

### DGE library sequencing and mapping sequences to the reference transcriptome database

Five DGE libraries were sequenced to generate 18.5-26.0 million clean reads per library after filtering the raw reads. The total number of mapped reads in each library ranged from 14.9 to 20.8 million, and the percentage of these reads ranged from 78.92 to 91.04%. The number of unique match reads ranged from 11.4 to 16.0 million (Table 1), and almost reached saturation above 8 million (Additional file 9A). We also evaluated the randomness of the DGE data by analyzing the distribution of reads by matching them to the reference genes [24]. Most reads were evenly distributed throughout the reference genes, suggesting that the randomness of the data was reasonable (Additional file 9B). The distribution of unique reads was used to evaluate the normality of the RNA-Seq data. Five RNA-Seq libraries showed similar distribution patterns of unique reads over different abundance categories with approximately 21-28% of the sequences having a similarity of 90% (Additional file 9C).

**Table 1:** Summary of read numbers based on the RNA-Seq data obtained from each sample

Map to gene	Reads number				
	0W	2W + V	2W-V	3W + V	3W-V
Total reads	19,519,570	21,999,628	19,615,348	18,455,548	26,001,726
Total base pairs	1,951,957,000	2,199,962,800	1,961,534,800	1,845,554,800	2,600,172,600
Total mapped reads	15,403,899	17,602,328	15,895,801	14,880,551	20,840,238
Perfect match	10,902,682	12,365,769	11,117,796	10,316,215	14,311,296
≤2 bp mismatch	4,501,217	5,236,559	4,778,005	4,564,336	6,528,942
Unique match	11,774,798	13,386,594	12,147,205	11,350,543	16,042,994
Multi-position match	3,629,101	4,215,734	3,748,596	3,530,008	4,797,244
Total unmapped reads	4,115,671	4,397,300	3,719,547	3,574,997	5,161,488

### Changes in gene expression profiles during floral induction

**Fig. 3:**
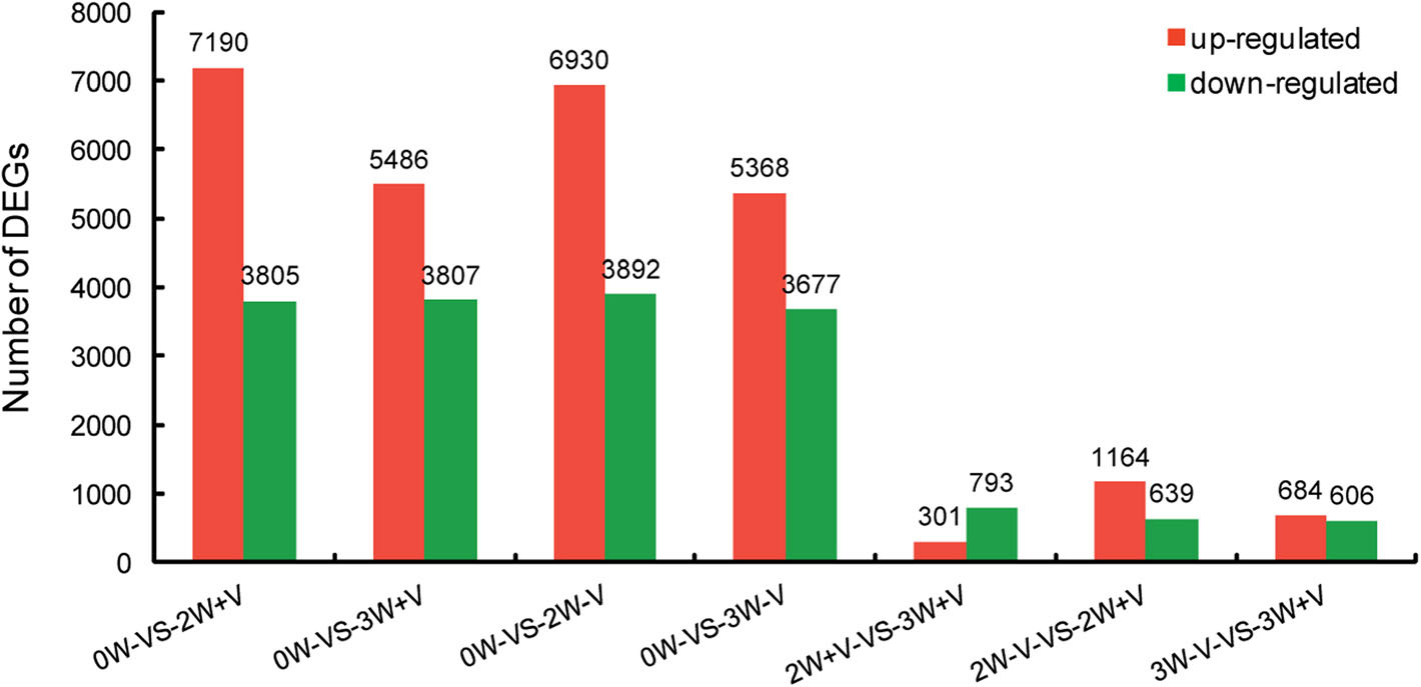
Effects of low temperature treatment on changes in gene expression profiles. The numbers of up- and down-regulated genes are shown in pairwise comparisons of the five libraries

Differences in gene expression during floral induction were examined, and DEGs were identified by pairwise comparisons of the five libraries, i.e. 0W-vs-2W + V, 0W-vs-3W + V, 0W-vs-2W-V, 0W-vs-3W-V, 2W + V-vs-3W + V, 2W-V-vs-2W + V and 3W-V-vs-3W + V, with |log_2_ Ratio| ≥ 1 as the threshold of expression fold and FDR ≤ 10^-5^ as the false discovery rate (Fig. 3). The greatest number of DEGs was found between the 0W and 2W + V libraries, and a total of 10,995 DEGs were detected, with 7190 up-regulated and 3805 down-regulated. This suggested that many changes in gene expression paralleled the low temperature treatment. However, the least number of DEGs occurred between the 2W + V and 3W + V libraries, indicating that changes in leaves had already occurred after 2 weeks of low temperature treatment. In addition, 1803 and 1290 genes were significantly differentially expressed between 2W-V-vs-2W + V and 3W-V-vs-3W + V.

### Functional classification of DEGs during floral induction

**Fig. 4:**
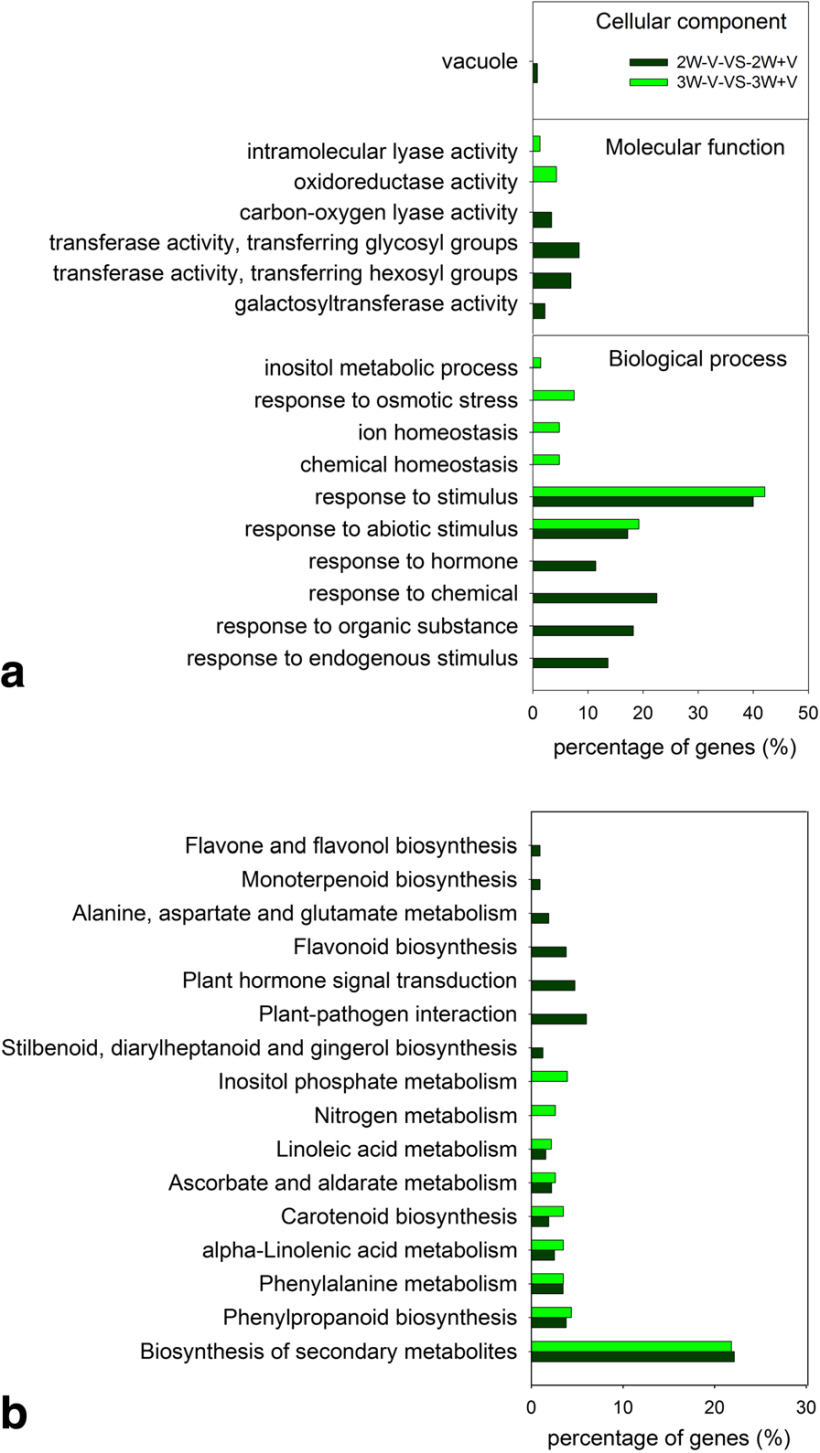
Function analysis of DEGs during floral induction based on Gene Ontology and KEGG pathway. GO category (**a**) and KEGG pathway (**b**) mRNAs that were significantly enriched were analyzed in pairwise comparisons, with the dark green for 2W-V-vs-2W + V and bright green for 3W-V-vs-3W + V. Percentages are calculated based on the proportion of DEGs in each set

In order to identify the major transcriptome changes during temperature-dependent floral induction in litchi, we performed studies of enrichment of DEGs in GO and KEGG functional categories in pairwise comparisons of 2W-V-vs-2W + V and 3W-V-vs-3W + V. GO functional enrichment showed that 17 GO terms changed significantly after 2 and 3 weeks of low temperature treatment (Fig. 4a). In the cellular component category, only ‘vacuole’ was significantly enriched in 2W-V-vs-2W + V. In the molecular function category, four GO terms ‘carbon-oxygen lyase activity’, ‘galactosyltransferase activity’, ‘transferase activity, transferring hexosyl groups’ and ‘transferase activity, transferring glycosyl groups’ were significantly enriched in 2W-V-vs-2W + V, while two GO term ‘oxidoreductase activity’ and ‘intramolecular lyase activity’ were highly enriched in 3W-V-vs-3W + V. In the biological process category, GO terms ‘response to abiotic stimulus’ and ‘response to stimulus’ were both enriched in two libraries, of which ‘response to stimulus’ accounted for the highest proportion. ‘Inositol metabolic process’, ‘response to osmotic stress’, ‘ion homeostasis’ and ‘chemical homeostasis’ were only enriched in 3W-V-vs-3W + V. ‘Response to hormone’, ‘response to chemical’, ‘response to organic substance’ and ‘response to endogenous stimulus’ were significantly enriched only in 2W-V-vs-2W + V (Fig. 4a).

In 2W-V-vs-2W + V and 3W-V-vs-3W + V comparisons, 316 and 229 DEGs mapped to 14 and 9 KEGG pathways, respectively. There were 7 KEGG pathways enriched in both 2W-V-vs-2W + V and 3W-V-vs-3W + V; most enrichment of unigenes in the two libraries involved sequences in the ‘Biosynthesis of secondary metabolites’ pathway. Of the 316 DEGs in the 2W-V-vs-2W + V comparison, 62 (19.6%) DEGs were mapped to 7 pathways, with specific enrichment of unigenes involved in ‘Plant-pathogen interaction’ and ‘Plant hormone signal transduction’. Comparing the 3W + V and 3W-V libraries, 15 (6.6%) DEGs were identified in two pathways, with those in the ‘Inositol phosphate metabolism’ and ‘Nitrogen metabolism’ pathway significantly enriched (Fig. 4b).

### Transcriptome changes during floral induction

**Fig. 5:**
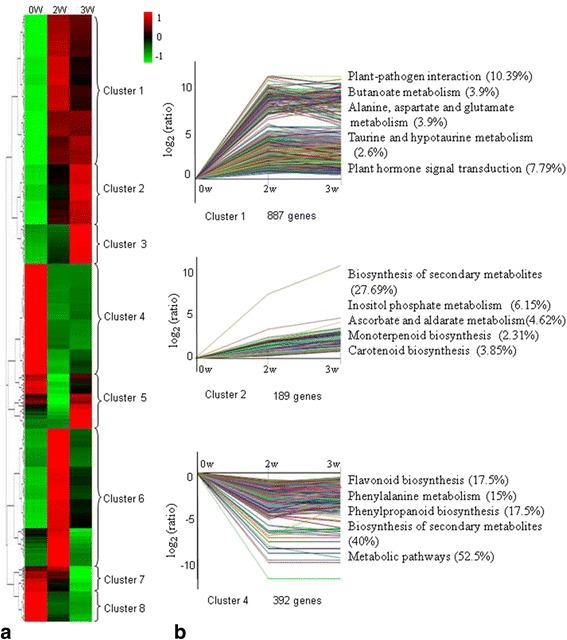
Cluster analysis of DGEs during floral induction. **a** Hierarchical clustering analysis of DGEs. The log_10_ (RPKM + 1) for significantly DEGs from the three selected stages (0, 2 and 3 weeks after low temperature treatment) was used. The 2755 DEGs were classified into 8 regulation patterns. The red and blue rectangles denoted the hierarchical clustering. **b** The significant expression profiles and their top 5 most significantly enriched functional pathways. The log_2_(ratios) for significantly DEGs from the three selected stages (0, 2 and 3 weeks after low temperature treatment) was used. The number at the bottom of a cluster box is the number of genes assigned to the profile. The number after each pathway represents the percentage of DEGs genes with annotation into this pathway

A total of 2755 DEGs were unioned and obtained by the pairwise comparisons of 2W-V-vs-2W + V and 3W-V-vs-3W + V. To understand the expression patterns of 2755 genes, cluster analyses of genes showing stage-specific expression were performed and results displayed in a heat map (Fig. 5a). Subsequently, we used the STEM software to summarize our filtered data, and 2,146 genes were divided into eight groups based on their expression modulation patterns (Additional file 10). The clustering analysis revealed three significant expression clusters (*p*-value ≤ 0.05) during floral induction, cluster 1, 2 and 4 (Fig. 5b, Additional file 10). Profile 1 included 887 unigenes that were up-regulated rapidly at 2 week after low temperature treatment. The 189 unigenes in Profile 2 had a similar expression pattern to those in Profile 1 but were expressed at a higher level at 3 week during floral induction. Significant pathways enriched in Profile 1were Plant-pathogen interaction (10.39%), Butanoate metabolism (3.9%), Alanine, aspartate and glutamate, metabolism (3.9%), Taurine and hypotaurine metabolism (2.6%), and Plant hormone signal transduction (7.79%). Top five most significantly enriched in Profile 2 were related to pathways like Biosynthesis of secondary metabolites (27.69%), Inositol phosphate metabolism (6.15%), Ascorbate and aldarate metabolism (4.62%), Monoterpenoid biosynthesis (2.31%), and Carotenoid biosynthesis (3.85%). The 392 unigenes in Profile 4 were most highly expressed at dormancy and reduced to low levels during floral induction. Top enriched pathways included Flavonoid biosynthesis (17.5%), Phenylalanine metabolism (15%), Phenylpropanoid biosynthesis (17.5%), Biosynthesis of secondary metabolites (40%), and Metabolic pathways (52.5%) (Fig. 5b).

To better search the key genes related to floral induction in litchi, 276 DEGs were further detected significantly differentially expressed under low temperature treatment, in the meantime, no significant changes were found in control treatment (Additional file 11). Among the 276 DEGs, some transcription factors including WD40 repeat-like superfamily protein, TCP, bHLH, GATA and MYB were found highly expressed. Genes related to sugar signals (galactinol synthase, galactose oxidase, 6-phosphofructokinase, trehalose-6-phosphate synthase and mannose/glucose-specific lectin) were also found in this group. More interestingly, one *FT* homologues gene (Unigene0025395) was found to be very obviously induced by low temperature (Additional file 11).

### Genes related to metabolism of different sugars during floral induction

**Fig. 6:**
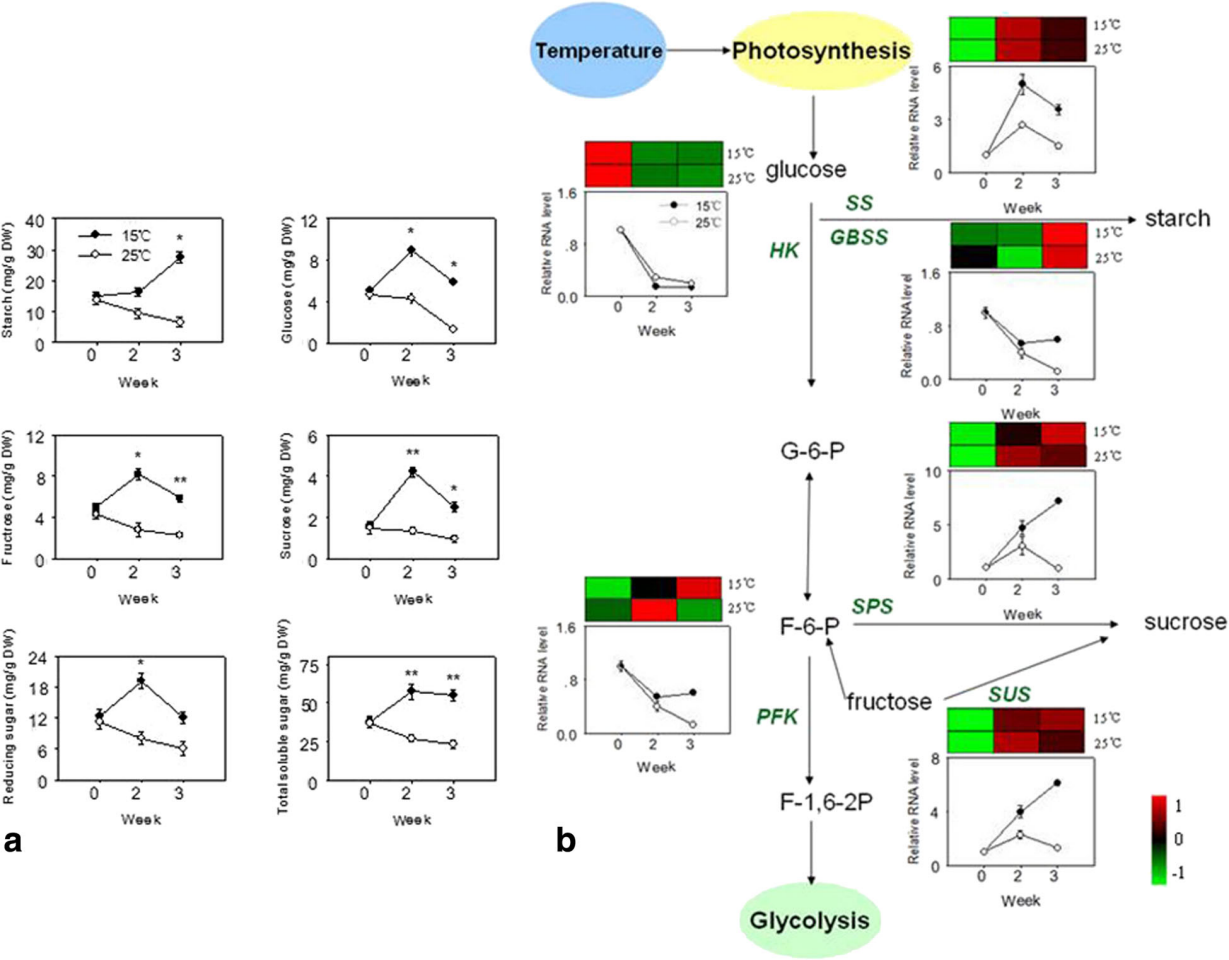
Concentrations of different sugars in litchi leaves during floral induction. **a** Dynamic changes in content of different sugars during low temperature induction. *Bars* represent the standard error (*n* = 3). * and ** for same time represented significant difference at 0.05 and 0.01 levels. **b** Simplified scheme of the sugar metabolism pathway. Key enzyme names in green color were abbreviated as follow: *HK*: *hexokinase*, *SS*: *starch synthase*, *GBSS*: *granule-bound starch synthase, PFK*: *phosphofructokinase*, *SPS*: *sucrose phosophate synthase*, *SUS*: *sucrose synthase.* The expression patterns are indicated on the side of each key gene, with the upside representing the relative log_10_ (RPKM + 1) respectively. The grids with different colors from *green* to *red* show the relative expression magnitude. Low temperature (15 °C, *black circles*) and the control (25 °C, *white circles*) leaves sampled at different time were used in the analysis

Since many pathways involved in carbohydrate metabolism were found according to KEGG enrichment analysis (Additional file 8), and the expression of a number of genes implicated in carbohydrate metabolism varied greatly during floral induction (Additional file 11), changes in sugars content were investigated. The concentrations of different sugars in leaves under low temperature treatment (15 °C) were significantly higher than those in control plants (25 °C) throughout floral induction (Fig. 6a). The concentration of starch increased markedly in the leaves under low temperature. The contents of glucose, fructose, sucrose, reducing sugar and total soluble sugar increased and reached a peak 2 weeks after low temperature treatment, and then rapidly decreased. In this study, the expression of some unigenes involved in sugar metabolism were further analyzed by RT-qPCR, *Starch synthase* (*SS*), *granule-bound starch synthase* (*GBSS*), *sucrose phosphate synthase* (*SPS*), *sucrose synthase* (*SUS*) and *phosphofructokinase* (*PFK*) significantly increased after low temperature induction, and these results were consistent with the data derived from RNA-Seq (Fig. 6b).

### Identification of flowering-related genes and analysis of variations in FT gene expression during floral induction

Four flowering regulatory pathways have been extensively investigated in *Arabidopsis* [55–57]. In order to identify flowering-related genes during litchi floral induction, BLAST against NCBI Nr protein datasets was performed, and we found at least 76 unigenes with homology to known flowering-related genes (Additional file 12). These identified flowering-related unigenes from litchi leaves are shown in Additional file 13. But we found no significant differences in the most genes between treatment and control, suggesting the presence and different function of these floral pathways in litchi. The discovery of these genes will help in future studies on the mechanism of floral induction in litchi. In order to verify the RNA-Seq results, RT-qPCR was used to measure the expression of 14 genes, which included transcription factors (Additional file 14) and floral genes. The RT-qPCR results were basically consistent with the RNA-Seq data from the five samples. Linear regression [(RNA-Seq value) = a (RT-qPCR value) + b] analysis showed a significant correlation (*R* ^2^ = 0.858), indicating a strong correlation between expression profile assayed by RT-qPCR and RNA-Seq (Additional file 14).

**Fig. 7:**
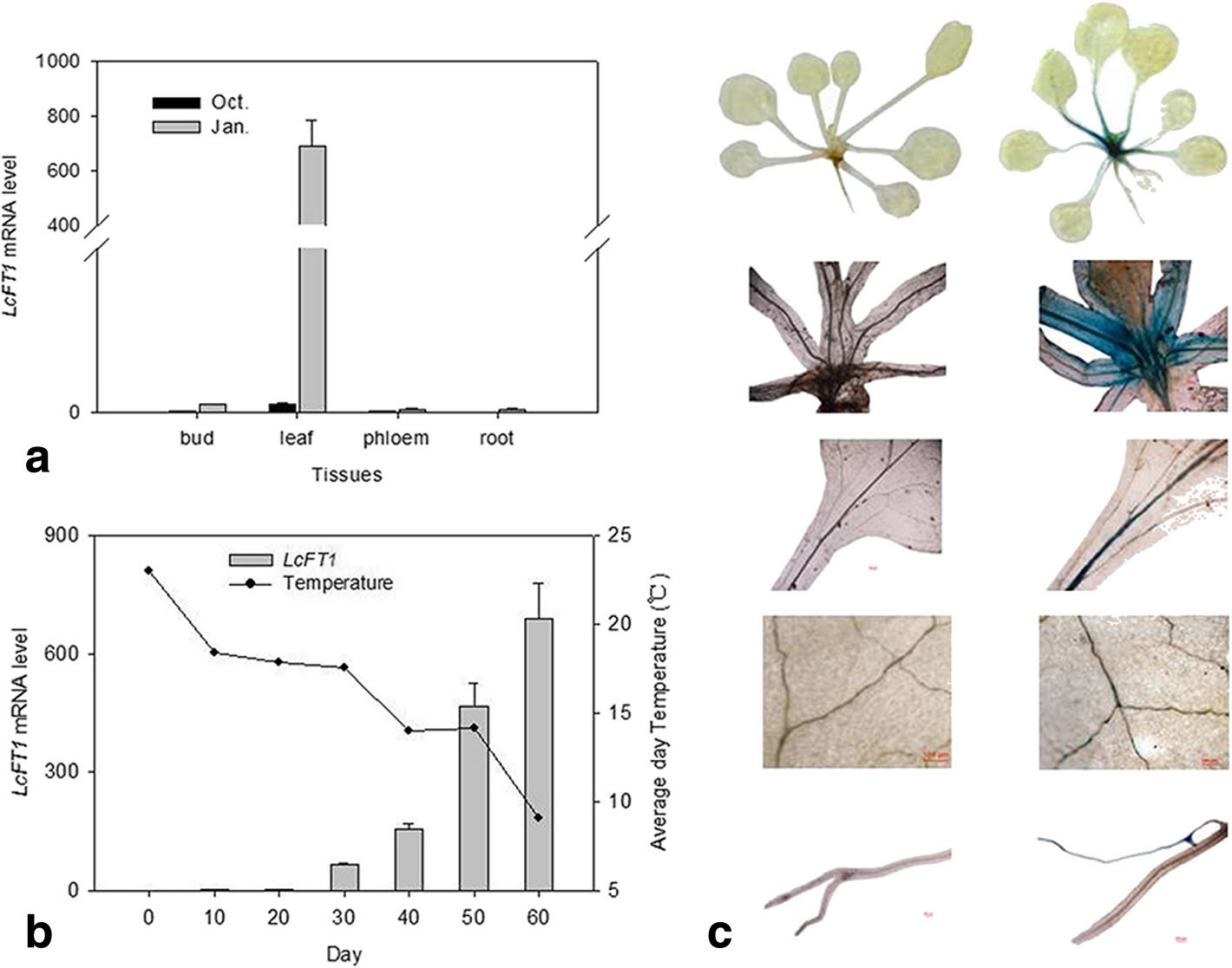
Expression analysis of *LcFT1* gene and GUS-staining of transgenic Arabidopsis. **a** Expression analysis of *LcFT1* gene in various organs. **b** Expression analysis of *LcFT1* gene during flower bud differentiation in leaf, and the change of temperature in field. **c** Comparison of GUS-staining pattern of transgenic Arabidopsis and its wild type after low temperature treament. GUS-staining pattern of seedlings and close-up views of different tissues in wild-type Columbia (WT) plants (*left*) and LcFT1pro:: GUS plants (*right*). Close-up views of GUS-staining patterns in different tissues of Arabidopsis: apical region, leaf, leaf vein and root (from top to bottom)

Among the floral genes, the transcripts of one *FT* gene (*Unigene0025396*) were dramatically up-regulated after low temperature induction according to RNA-Seq database, and this result was consistent with data derived from RT-qPCR (Additional file 14). Thus, the key *FT* homologous gene (*Unigene0025396*) was isolated from the transcriptome database. The *FT* gene encodes a protein called “florigen”, which has been shown to be a promoter of plant flowering [20–23, 58]. We performed further bioinformatics analysis of this gene sequences with multiple sequence alignment and phylogeny tree analysis (Additional file 15), and found that *Unigene0025396* had the same homologous with *LcFT1*, which is a leading cause of flowering timing in litchi and played a pivotal role in litchi floral induction by low-temperature [59]. Further results of the real-time quantitative expression analysis showed that the *LcFT1* gene exhibited apparent tissue specificity and that is only expressed in mature leaves of January. And the expression trend of LcFT1 gene in leaf was contrary to the change of temperature in field during floral differentiation (Fig. 7a, b). To better understand the molecular function of *LcFT1* gene, we obtained 2065 bp promoter sequences of *LcFT1* from the whole litchi genome sequencing, and found a number of cis-acting elements by the online software of Plant CARE, in which two LTR cis-acting elements involved in low-temperature responsiveness (Additional file 16). The results of GUS staining of transgenic Arabidopsis also showed strong GUS staining was observed in the leaves, petioles and roots in the plants of LcFT1pro::GUS with low temperature-treated.

## Discussion

### Changes in de novo assembled litchi leaf transcriptome in response to low temperature treatment

In litchi there is a strong requirement for exposure to low temperature for floral induction [3–5]. In the present study, a few enclosed litchi leaves were exposed to low temperature and the remaining leaves on other branches of the same tree were used as controls, and the leaves were then sampled at different times to investigate the changes in candidate genes during litchi floral induction. Recently, next generation sequencing has been widely used for profiling transcriptomic datasets to reveal the mechanisms involved in a range of biological events for non-model species [27–29]. To monitor the transcriptional changes in litchi leaves during low temperature-induced floral induction, five DGE libraries (0W, 2W + V, 3W + V, 2W-V and 3W-V) were constructed and from these 83,107 unigenes were assembled with a mean length of 1221 bp, which were much longer than previous studies [30, 32, 33]. These results demonstrated that RNA-Seq is an effective way to obtain a large portion of the transcriptome from a specific tissue. 51,448 (60.58%) unigenes were annotated using four public databases, indicating that the remaining 39.42% of sequences have no hit to the database, and thus new functions may be identified for these new genes in the future.

Through RNA-Seq analysis, we found that a total of 10,995, 6263 and 1094 genes were differentially expressed between 0W and 2W + V, 0W and 3W + V, as well as 2W + V and 3W + V. These results showed that the numbers of DEGs were lower after 3 weeks compared to 2 weeks treatment. This could be explained if most genes respond rapidly to the low temperature, but some other genes require a longer time of low temperature to activate or repress their expression, as with other cold responses [60]. DEGs derived from comparison of 0W-vs-2W-V and 0W-vs-3W-V libraries implied lots of gene expression changes occurred during leaf development under normal temperature and the control (2W-V vs 3W-V) in this study enabled these genes to be eliminated. Therefore, a significant proportion of the DEGs identified in the 2W-V-vs-2W + V and 3W-V-vs-3W + V comparisons are likely to be closely related to floral induction of litchi, and not simply involved in development or physiological responses to cold.

### Candidate genes involved in sugar metabolism during floral induction

Plants integrate diverse environmental and endogenous signals to ensure the timely transition from vegetative growth to flowering, which is a consuming energy process while the energy mainly comes from carbohydrates. Carbohydrates are thought to play a crucial role in the regulation of flowering, and many previous studies suggested the content of carbohydrates is correlated with floral formation in some horticultural plants [13, 17, 61–63]. Recently, trehalose-6-phosphate (T6P) has been suggested to function as a proxy for carbohydrate status in plants, *trehalose-6-phosphate synthase 1* (*TPS1*) is essential for normal vegetative growth and transition to flowering in Arabidopsis, the loss of *TPS1* causes Arabidopsis thaliana to flower extremely late, even under otherwise inductive environmental conditions [64, 65]. Wahl et al. [65] also found TPS1 activity is required for the induction of the florigen FT in the leaves, even under inductive photoperiod. In the present study, many genes related to sugar signals, such as galactinol synthase, galactose oxidase, 6-phosphofructokinase, trehalose-6-phosphate synthase and mannose/glucose-specific lectin, were also detected significantly differentially expressed under low temperature treatment. Simultaneously, the concentrations of carbohydrates accumulated paralleled increases during floral induction in expression of related biosynthesis genes such as *SS*, *GBSS*, *SPS*, and *SUS.* Similar results have also been found during flower induction in *Doritaenopsis* [13]. Additionally, sugar signals were also found to function as primary messengers during transition of plant vegetative growth into reproductive stage [66]. However, the fluctuating patterns of sucrose and starch found in this study indicate their metabolism is complex and they may play different roles at different stages. Thus, it is still needs more study that whether the sugar metabolism pathway acts as a signal that coordinates the induction of flowering by sensing the low temperature and regulating the expression of key floral integrators in leaves.

### Candidate genes involved in flowering pathways during floral induction

Seventy six flowering homologues to genes in four major flowering pathways were identified in our study, suggesting that all of the four flowering pathways may be present in litchi as well (Additional file 12). This was consistent with the findings of other transcriptomic analyses in litchi [18]. However, no significant difference of most flowering-related genes needed further study to investigate their exact functions in litchi. Low temperature is considered to be crucial cue for litchi flowering [2–6]. Thus we focused on the putative orthologues involved in the vernalization pathway, but in the present study, we found no significant differences in the expression of homologues of these genes between treatment and control, which possibly indicated a different mechanism of low temperature induced floral transition in litchi compared with vernalization in *Arabidopsis* (Additional file 13). We detected one *FT* homologue (*Unigene0025396*) in litchi leaves, and RNA-Seq data showed that the transcript level of *Unigene0025396* was up-regulated 12-fold during floral induction. FT protein is universally considered as “florigen” in plants [19–21], and epigenetic overexpression of *LcFT1* in *Arabidopsis* and tobacco can cause extremely early flowering [39]. In agreement with the expression pattern of *CiFT* in leaves of citrus, which increased when the temperature decreased [12], our results also showed that the *LcFT1* gene exhibited an apparent specific tissue in leaves, and the expression trend of LcFT1 gene was contrary to the change of temperature (Fig. 7a, b). The results of GUS staining of LcFT1pro::GUS transgenic Arabidopsis also showed GUS activity driven by *LcFT1* gene promoter can be induced by low temperature. All these results showed that *LcFT1* gene is closely related to the low temperature, and it may play a bridge and link in the relationship between induction of flowering and sensing the low temperature in leaves.

## Conclusions

This study provides a global survey of transcriptomes from litchi leaves exposed to low temperature of different time through de novo assembly of next generation sequencing technique. A total of 2755 differentially expressed genes were identified in response to low temperature. The expression levels of most genes of sugar metabolism pathway were found co-ordinated regulated coincident with the accumulation of sugars. The candidate *FT* gene that likely regulate flowering of litchi were also identified, and its possible role in the litchi leaves under low temperature induction flower were discussed. This study provides a platform for understanding the molecular mechanisms underlying changes in response to low temperature treatment in leaves, and will benefit researches in flowering mechanisms of evergreen fruit trees.

## Additional files

Additional file 1: The diurnal change of *LcFT1* expression. The relative RT-qPCR expression level of *LcFT1* was shown on the y-axis, and the sampled time was indicated on the x-axis. Actin was used as the internal control. The color white represented day, gray stood for night. Bars represent the standard error (*n* = 3). (PDF 30 kb)

Additional file 2: Schematics of the transcriptome sequencing analysis in litchi. (A) Experiment pipeline of transcriptome. Beads with Oligo(dT) are used to isolate poly(A) mRNA after total RNA is collected from eukaryote. Fragmentation buffer is added for interrupting mRNA to short fragments. Taking these short fragments as templates, random hexamer-primer is used to synthesize the first-strand cDNA. The second-strand cDNA is synthesized using buffer, dNTPs, RNaseH and DNA polymerase I, respectively. Short fragments are purified with QiaQuick PCR extraction kit and resolved with EB buffer for end reparation and adding poly (A). After that, the short fragments are connected with sequencing adapters. And, after the agarose gel electrophoresis, the suitable fragments are selected for the PCR amplification as templates. At last, the library could be sequenced using Illumina HiSeq™ 2500. (B) Workflow of the data assembly. Transcriptome de novo assembly is carried out with short reads assembling program-Trinity. Trinity firstly combines reads with certain length of overlap to form longer fragments without N, which are called contigs. Then, these contigs will be taken into further process of sequence cluster with sequence clustering software to form longer sequences without N, Such sequences are defined as Unigenes. When multiple samples from a same species are sequenced, Unigenes from each sample’s assembly can be taken into further process of sequence splicing and redundancy removing with sequence clustering software to acquire non-redundant Unigenes as long as possible. (PDF 120 kb)

Additional file 3: Primers of candidate and reference genes used for RT-qPCR. (PDF 90 kb)

Additional file 4: Summary statistics of the sequence assembly. (PDF 31 kb)

Additional file 5: Length distribution and number of Contigs (A) and Unigenes (B) assembly. The horizontal coordinates are lengths and the vertical coordinates are numbers of Contigs and Unigenes. (PDF 19 kb)

Additional file 6: Histogram of GO classifications for litchi leaf transcriptome. The unigenes were assigned to three main categories: biological process, cellular component, and molecular function. The left and right-hand y-axes indicate the percentage and number of annotated unigenes respectively. (PDF 224 kb)

Additional file 7: COG functional classifications of the litchi leaf transcriptome. COG is a database where orthologous gene products are classified. Every protein in COG is assumed to evolve from an ancestor protein, and the whole database is built on coding proteins with complete genome as well as system evolution relationships of bacteria, algae and eukaryotic creatures. Unigenes are aligned to COG database to predict and classify possible functions of Unigenes. A total of 18,497 unigenes were classified into 25 COG categories. (PDF 54 kb)

Additional file 8: Pathway assignment based on KEGG. (PDF 24 kb)

Additional file 9: Sequencing Assessment. (A) Sequencing saturation analysis result; (B) Distribution statistics of reads mapped to reference gene; (C) Gene coverage statistics. (PDF 176 kb)

Additional file 10: All STEM profiles by significance. Profiles ordered based on the *p*-value significance of number of genes were assigned versus expected. The ID and gene numbers of profiles were showed on the top of frames. *P*-values were represented with the number inside. Colored figures denoted the *p*-value (*p* < 0.05) significance, and the similar trends were set with the same color. (PDF 104 kb)

Additional file 11: Hierarchical clustering analysis and category distribution of DGEs during floral induction. 276 modulated DEGs were obtained from the gene expression profiles of only significantly differentially expressed under low temperature treatment, but no significant change in control treatment. The RPKM for significantly DEGs was used, the up-regulated and down-regulated genes are indicated in red and green respectively. The grids with different ranks from left to right showed 0W, 2W + V, 3W + V, 2W-V and 3W-V. (PDF 247 kb)

Additional file 12: Simplified diagram showing the four major genetic pathways regulating the floral transition in Arabidopsis (Boss et al. 2004; Putterill et al. 2004). Arrows indicate activation and straight lines ending with a perpendicular line indicate repression. The numbers below each gene represent the members of the corresponding genes found from RNA-Seq data for litchi. (PDF 129 kb)

Additional file 13: Putative orthologs of litchi genes involved in flowering induction. (PDF 139 kb)

Additional file 14: RT-qPCR verification of differential expression. The figure shows transcript levels of 14 genes, of which 6 probably associated with transcription factors (A) and 8 with flowering-related genes (B) under 15 °C (black) and 25 °C (white) treatment by comparing of RT-qPCR data (bar) with RNA-seq data (line). The relative RT-qPCR expression level is shown on the y-axis to the left, bars represent the standard error (*n* = 3), and the normalized expression level (RPKM) of RNA-seq is indicated on the y-axis to the right. Actin was used as the internal control. (C) Coefficient analysis between gene expression ratios obtained from RNA-seq data and RT-qPCR. The RT-qPCR log_2_ expression ratios (x-axis) were plotted against RNA-seq data ratios (y-axis). (PDF 160 kb)

Additional file 15: Analysis of FT homologous transcripts in litchi. (A) Alignment of the FT homologues proteins. (B) Phylogenetic relationship between Unigene0025396 and other FT proteins from higher plant species. The accession numbers of the sequences used for the alignment are: LcFT1 [*Litchi chinensis*, AEU08960.1], LcFT2 [*Litchi chinensis*, AEU08961.1], DlFT1 [*Dimocarpus longan*, AEZ63949.1], DlFT2 [*Dimocarpus longan*, AEZ63950.1], MdFT1 [*Malus* x *domestica*, BAD08340.1], VvFT1 [*Vitis vinifera*, ABL98120.1], AtFT [*Arabidopsis thaliana*, BAA77838], CuFT [*Citrus unshiu*, BAF96644.1], PtFT [*Populus tomentosa*, AFU08239.1] and GmFT [*Glycine max*, BAJ33489.1]. (PDF 231 kb)

Additional file 16: The analysis of the *LcFT1* promoter elements. (PDF 243 kb)

## Abbreviations

COG: Clusters of Orthologous Groups
DEGs: Differentially-expressed genes
DGE: Digital gene expression
*FT*: *Flowering locus T*
*GBSS*: *Granule-bound starch synthase*
GO: Gene ontology
*HK*: *Hexokinase*
KEGG: Kyoto Encyclopedia of Genes and Genomes
Nr: NCBI non-redundant database
*PFK*: *Phosphofructokinase*
RNA-Seq: RNA sequencing
RPKM: Read per kilobase per million
RT-qPCR: Reverse transcription, quantitative polymerase chain reaction
*SPS*: *Sucrose phosphate synthase*
*SS*: *Starch synthase*
*SUS*: *Sucrose synthase*
Swiss-Prot: Swiss-Prot protein database

## References

[1] Huang HB, Chen HB (2003). A phase approach towards floral formation in lychee. Acta Hort.

[2] Menzel CM, Paxton BF (1985). The effect of temperature on growth and dry matter production of lychee seedlings. Sci Hortic.

[3] Menzel CM, Simpson DR (1988). Effect of temperature on growth and flowering of litchi (*Litchi chinensis* Sonn.) cultivars. J Hortic Sci Biotech.

[4] Menzel CM, Rasmussen TS, Simpson DR (1989). Effects of temperature and leaf water stress on growth and flowering of litchi (*Litchi chinensis* Sonn.). J Hortic Sci Biotech.

[5] Batten DJ, Mcconchie CA (1995). Floral induction in growing buds of lychee (*Litchi chinensis*) and mango (*Mangifera indica*). Aust J Plant Physiol.

[6] Chen HB, Huang HB (2005). Low temperature requirements for floral induction in lychee. Acta Hort.

[7] Hartmann HT, Fadl MS, Hackett WP (1967). Initiation of flowering and changes in endogenous inhibitors and promotors in olive buds as a result of chilling. Physiol Plant.

[8] Whiley AW, Rasmussen TS, Saranah JB, Wolstenholme BN (1989). Effect of temperature on growth, dry matter production and starch accumulation in ten mango (*Mangifera indica* L.) cultivars. J Hortic Sci Biotech.

[9] Chaikiattiyos S, Menzel CM, Rasmussen TS (1994). Floral induction in tropical fruit trees: effects of temperature and water supply. J Hortic Sci Biotech.

[10] Southwick SM, Davenport TL (1986). Characterization of water stress and low temperature effects on flower induction in citrus. Plant Physiol.

[11] Davenport TL (1990). Citrus flowering. Hortic Rev.

[12] Nishikawa F, Endo T, Shimada T, Fujii H, Shimizu T, Omura M (2007). Increased *CiFT* abundance in the stem correlates with floral induction by low temperature in Satsuma mandarin (*Citrus unshiu* Marc.). J Exp Bot.

[13] Qin QP, Quentin K, Zhang C, Zhou LP, Luo XY, Zhou MB (2012). The cold awakening of *Doritaenopsis* ‘Tinny Tender’ orchid flowers: the role of leaves in cold-induced bud dormancy release. J Plant Growth Regul.

[14] Chen HB, Huang HB (2000). China litchi industry: development, achievements and problems. Acta Hort.

[15] Dubois A, Remay A, Raymond O, Balzergue S, Chauvet A, Maene M (2011). Genomic approach to study floral development genes in *Rosa* sp. PLoS One.

[16] Zhang XM, Zhao L, Larson-Rabin Z, Li DZ, Guo ZH (2012). *De novo* sequencing and characterization of the floral transcriptome of *Dendrocalamus latiflorus* (Poaceae: Bambusoideae). PLoS One.

[17] Zhang D, Ren L, Yue JH, Wang L, Zhuo LH, Shen XH (2013). A comprehensive analysis of flowering transition in *Agapanthus praecox* ssp orientalis (Leighton) Leighton by using transcriptomic and proteomic techniques. J Proteomics.

[18] Zhang HN, Wei YZ, Shen JY, Lai B, Huang XM, Ding F (2014). Transcriptomic analysis of floral initiation in litchi (*Litchi chinensis* Sonn.) based on de novo RNA sequencing. Plant Cell Rep.

[19] Corbesier L, Vincent C, Jang S, Fornara F, Fan Q, Searle I (2007). FT protein movement contributes to long-distance signaling in floral induction of *Arabidopsis*. Science.

[20] Zeevaart JA (2008). Leaf-produced floral signals. Curr Opin Plant Biol.

[21] Kotoda N, Hayashi H, Suzuki M, Igarashi M, Hatsuyama Y, Kidou S (2010). Molecular characterization of *FLOWERING LOCUS T-*like genes of apple (*Malus* x *domestica* Borkh.). Plant Cell Physiol.

[22] Lin MK, Belanger H, Lee YJ, Varkonyi-Gasic E, Taoka K, Miura E (2007). FLOWERING LOCUS T protein may act as the long-distance florigenic signal in the cucurbits. Plant Cell.

[23] Mathieu J, Warthmann N, Kuttner F, Schmid M (2007). Export of FT protein from phloem companion cells is sufficient for floral induction in *Arabidopsis*. Curr Biol.

[24] Wang Z, Gerstein M, Snyder M (2009). RNA-Seq: a revolutionary tool for transcriptomics. Nat Rev Genet.

[25] Surget-Groba Y, Montoya-Burgos JI (2010). Optimization of de novo transcriptome assembly from next-generation sequencing data. Genome Res.

[26] Xia ZH, Xu HM, Zhai JL, Li DJ, Luo HL, He CZ (2011). RNA-Seq analysis and de novo transcriptome assembly of *Hevea brasiliensis*. Plant Mol Biol.

[27] Liu GQ, Li WS, Zheng PH, Xu T, Chen LJ, Liu DF (2012). Transcriptomic analysis of ‘Suli’ pear (*Pyrus pyrifolia* white pear group) buds during the dormancy by RNA-Seq. BMC Genomics.

[28] Wang Y, Tao X, Tang XM, Xiao L, Sun JL, Yan XF (2013). Comparative transcriptome analysis of tomato (*Solanum lycopersicum*) in response to exogenous abscisic acid. BMC Genomics.

[29] Bai SL, Saito T, Sakamoto D, Ito A, Fujii H, Takaya M (2013). Transcriptome analysis of Japanese Pear (*Pyrus pyrifolia* Nakai) flower buds transitioning through endodormancy. Plant Cell Physiol.

[30] Feng C, Chen M, Xu CJ, Bai L, Yin XR, Li X (2012). Transcriptomic analysis of Chinese bayberry (*Myrica rubra*) fruit development and ripening using RNA-Seq. BMC Genomics.

[31] Yin YX, Zhang XW, Fang YJ, Pan LL, Sun GY, Xin CQ (2012). High-throughput sequencing-based gene profiling on multi-staged fruit development of date palm (*Phoenix dactylifera*, L.). Plant Mol Biol.

[32] Xie M, Huang Y, Zhang YP, Wang X, Yang H, Yu O (2013). Transcriptome profiling of fruit development and maturation in Chinese white pear (*Pyrus bretschneideri* Rehd). BMC Genomics.

[33] Li CQ, Wang Y, Huang XM, Li J, Wang HC, Li JG (2013). *De novo* assembly and characterization of fruit transcriptome in *Litchi chinensis* Sonn and analysis of differentially regulated genes in fruit in response to shading. BMC Genomics.

[34] Corbacho J, Romojaro F, Pech JC, Latche A, Gomez-Jimenez MC (2013). Transcriptomic events involved in melon mature-fruit abscission comprise the sequential induction of cell-wall degrading genes coupled to a stimulation of endo and exocytosis. PLoS One.

[35] Yamashino T, Yamawaki S, Hagui E, Ueoka-Nakanishi H, Nakamichi N, Ito S (2013). Clock-controlled and FLOWERING LOCUS T (FT)-dependent photoperiodic pathway in *Lotus japonicus* I: verification of the flowering-associated function of an FT homolog. Biosci Biotechnol Biochem.

[36] Shim JS, Imaizumi T (2015). Circadian clock and photoperiodic response in *Arabidopsis*: From seasonal flowering to redox homeostasis. Biochemistry.

[37] Zenoni S, Ferrarini A, Giacomelli E, Xumerle L, Fasoli M, Malerba G (2010). Characterization of transcriptional complexity during berry development in *Vitis vinifera* using RNA-Seq. Plant Physiol.

[38] Grabherr MG, Haas BJ, Yassour M, Levin JZ, Thompson DA, Amit I, Adiconis X (2011). Full-length transcriptome assembly from RNA-Seq data without a reference genome. Nat Biotechnol.

[39] Pertea G, Huang X, Liang F, Antonescu V, Sultana R, Karamycheva S (2003). TIGR Gene Indices clustering tools (TGICL): a software system for fast clustering of large EST datasets. Bioinformatics.

[40] Kanehisa M, Araki M, Goto S, Hattori M, Hirakawa M, Itoh M (2008). KEGG for linking genomes to life and the environment. Nucleic Acids Res.

[41] Mortazavi A, Williams BA, McCue K, Schaeffer L, Wold B (2008). Mapping and quantifying mammalian transcriptomes by RNA-Seq. Nat Methods.

[42] Robinson MD, McCarthy DJ, Smyth GK (2010). edgeR: a Bioconductor package for differential expression analysis of digital gene expression data. Bioinformatics.

[43] Conesa A, Gotz S, Garcia-Gomez JM, Terol J, Talon M, Robles M (2005). Blast2GO: a universal tool for annotation, visualization and analysis in functional genomics research. Bioinformatics.

[44] Ye J, Fang L, Zheng HK, Zhang Y, Chen J, Zhang ZJ (2006). WEGO: a web tool for plotting GO annotations. Nucleic Acids Res.

[45] Kanehisa M, Goto S (2000). KEGG: Kyoto Encyclopedia of Genes and Genomes. Nucleic Acids Res.

[46] Ernst J, Bar-Joseph Z (2006). STEM: a tool for the analysis of short time series gene expression data. BMC Bioinformatics.

[47] Zhang HF, Li H, Lai B, Xia HQ, Wang HC, Huang XM (2016). Morphological characterization and gene expression profiling during bud development in a tropical perennial, *Litchi chinensis* Sonn. Front Plant Sci.

[48] Wang TD, Zhang H F, Wu ZC, Li JG, Huang XM, Wang HC. Sugar uptake in the aril of litchi fruit depends on the apoplasmic post-phloem transport and the activity of proton pumps and the putative transporter LcSUT4. Plant and Cell Physiology. 2015;56(2):377-87. 10.1093/pcp/pcu173 25432972

[49] Wang HC, Huang HB, Huang XM, Hu ZQ (2006). Sugar and acid compositions in the arils of Litchi chinensis Sonn.: cultivar differences and evidence for the absence of succinic acid. J Hortic Sci Biotechnol.

[50] Sato KT, Richardson A, Timm DE, Sato K (1988). One-step iodine starch method for direct visualization of sweating. The American journal of the medical sciences.

[51] Livak KJ, Schmittgen TD (2001). Analysis of relative gene expression data using real-time quantitative PCR and the 2^-ΔΔCT^ method. Methods.

[52] Zhong HY, Chen JW, Li CQ, Chen L, Wu JY, Chen JY (2010). Selection of reliable reference genes for expression studies by reverse transcription quantitative real-time PCR in litchi under different experimental conditions. Plant Cell Rep.

[53] Zhang X, Henriques R, Lin SS, Niu QW, Chua NH (2006). *Agrobacterium*-mediated transformation of *Arabidopsis thaliana* using the floral dip method. Nat Protoc.

[54] Jefferson RA (1987). Assaying chimeric genes in plants: The GUS gene fusion system. Plant Mol Biol Rep.

[55] Boss PK, Bastow RM, Mylne JS, Dean C (2004). Multiple pathways in the decision to flower: enabling, promoting, and resetting. Plant Cell.

[56] Jack T (2004). Molecular and genetic mechanisms of floral control. Plant Cell.

[57] Putterill J, Laurie R, Macknight R (2004). It’s time to flower: the genetic control of flowering time. Bioessays.

[58] Jaeger KE, Wigge PA (2007). FT protein acts as a long-range signal in *Arabidopsis*. Curr Biol.

[59] Ding F, Zhang SW, Chen HB, Su ZX, Zhang R, Xiao QS (2015). Promoter difference of *LcFT1* is a leading cause of natural variation of flowering timing in different litchi cultivars (*Litchi chinensis* Sonn.). Plant Sci.

[60] Winfield MO, Lu C, Wilson ID, Coghill JA, Edwards KJ (2010). Plant responses to cold: transcriptome analysis of wheat. Plant Biotechnol J.

[61] Goldschmidt EE, Aschkenaki N, Herzano Y, Schaffer AA, Monselise SP (1985). A role for carbohydrate levels in the control of flowering in citrus. Sci Hortic.

[62] Menzel CM, Simpson DR (1987). Effect of cincturing on growth and flowering of lychee over several seasons in subtropical Queensland. Aust J Exp Agric.

[63] Chen HB, Huang HB, Liu ZL (2004). Flower formation and patterns of carbohydrate distribution in litchi trees. Acta Horticulturae Sinica.

[64] Van Dijken AJ, Schluepmann H, Smeekens S (2004). *Arabidopsis* trehalose-6-phosphate synthase 1 is essential for normal vegetative growth and transition to flowering. Plant Physiol.

[65] Wahl V, Ponnu J, Schlereth A, Arrivault S, Langenecker T, Franke A (2013). Regulation of flowering by trehalose-6-phosphate signaling in *Arabidopsis thaliana*. Science.

[66] Rolland F, Moore B, Sheen J (2002). Sugar sensing and signaling in plants. Plant Cell.

