# Supplementary material for: Transcriptome profiling of litchi leaves in response to low temperature reveals candidate regulatory genes and key metabolic events during floral induction
Source: BMC Genomics. 2017 May 10;18:363. doi: 10.1186/s12864-017-3747-x (PMC5424310; doi:10.1186/s12864-017-3747-x)
Supplement: Supplementary file 1 — The diurnal change of LcFT1 expression. The relative RT-qPCR expression level of LcFT1 was shown on the y-axis, and the sampled time was indicated on the x-axis. Actin was used as the internal control. The color white represented day, gray stood for night. Bars represent the standard error (n = 3). (PDF 30 kb) [file 12864_2017_3747_MOESM1_ESM.pdf]

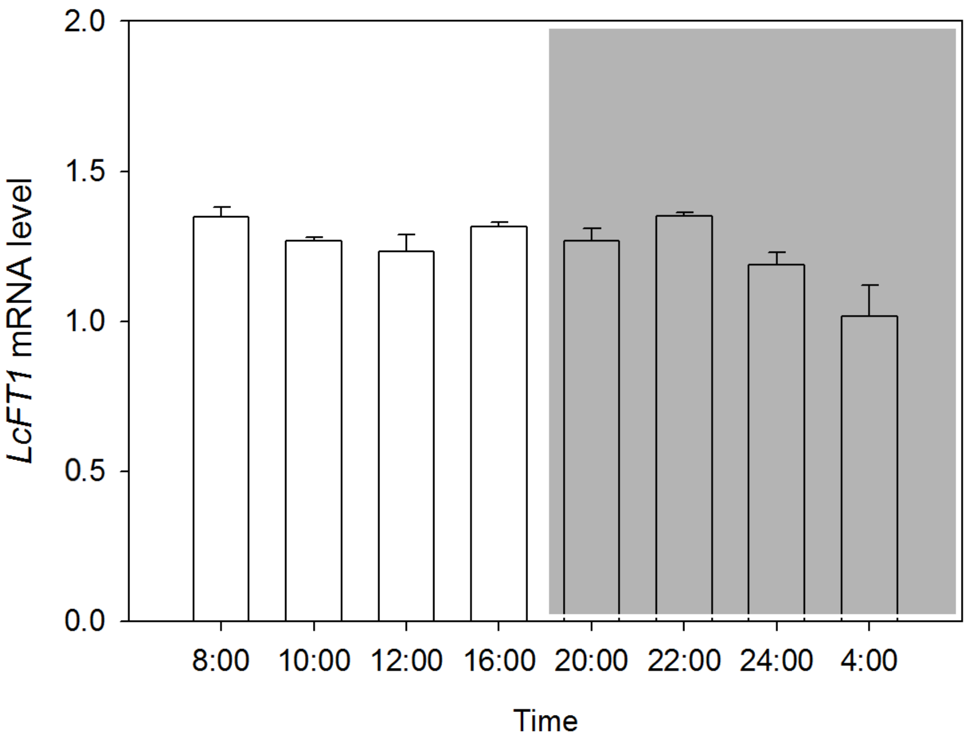

Additional file 1. The diurnal change of *LcFT1* expression.

The relative RT-qPCR expression level of *LcFT1* was shown on the y-axis, and the sampled time was indicated on the x-axis. Actin was used as the internal control. The color white represented day, gray stood for night. Bars represent the standard error (n=3).
