# Supplementary material for: Transcriptome profiling of litchi leaves in response to low temperature reveals candidate regulatory genes and key metabolic events during floral induction
Source: BMC Genomics. 2017 May 10;18:363. doi: 10.1186/s12864-017-3747-x (PMC5424310; doi:10.1186/s12864-017-3747-x)
Supplement: Supplementary file 2 — Schematics of the transcriptome sequencing analysis in litchi. (A) Experiment pipeline of transcriptome. Beads with Oligo(dT) are used to isolate poly(A) mRNA after total RNA is collected from eukaryote. Fragmentation buffer is added for interrupting mRNA to short fragments. Taking these short fragments as templates, random hexamer-primer is used to synthesize the first-strand cDNA. The second-strand cDNA is synthesized using buffer, dNTPs, RNaseH and DNA polymerase I, respectively. Short fragments are purified with QiaQuick PCR extraction kit and resolved with EB buffer for end reparation and adding poly (A). After that, the short fragments are connected with sequencing adapters. And, after the agarose gel electrophoresis, the suitable fragments are selected for the PCR amplification as templates. At last, the library could be sequenced using Illumina HiSeq™ 2500. (B) Workflow of the data assembly. Transcriptome de novo assembly is carried out with short reads assembling program-Trinity. Trinity firstly combines reads with certain length of overlap to form longer fragments without N, which are called contigs. Then, these contigs will be taken into further process of sequence cluster with sequence clustering software to form longer sequences without N, Such sequences are defined as Unigenes. When multiple samples from a same species are sequenced, Unigenes from each sample’s assembly can be taken into further process of sequence splicing and redundancy removing with sequence clustering software to acquire non-redundant Unigenes as long as possible. (PDF 120 kb) [file 12864_2017_3747_MOESM2_ESM.pdf]

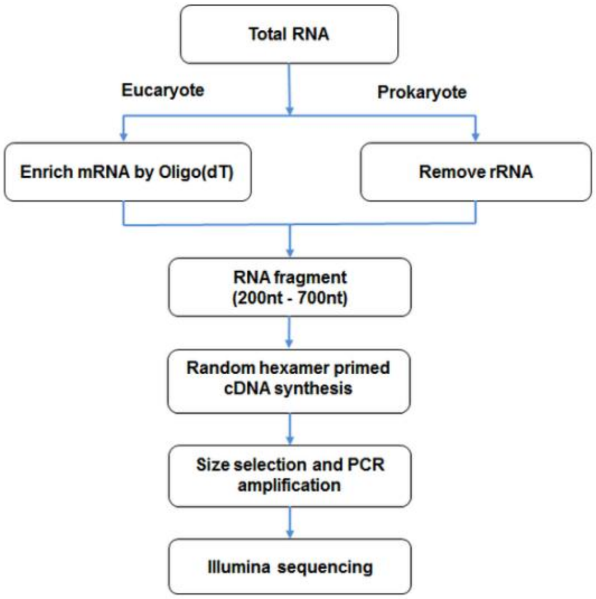


A

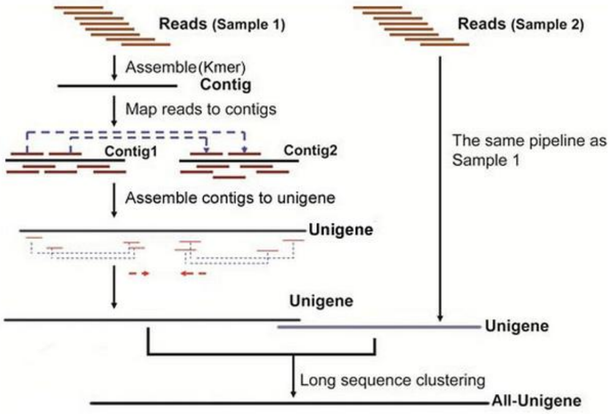


B

**Additional file 2. Schematics of the transcriptome sequencing analysis in litchi.**

(A) Experiment pipeline of transcriptome. Beads with Oligo(dT) are used to isolate poly(A) mRNA after total RNA is collected from eukaryote. Fragmentation buffer is added for interrupting mRNA to short fragments. Taking these short fragments as templates, random hexamer-primer is used to synthesize the first-strand cDNA. The second-strand cDNA is synthesized using buffer, dNTPs, RNaseH and DNA polymerase I, respectively. Short fragments are purified with QiaQuick PCR extraction kit and resolved with EB buffer for end reparation and adding poly(A). After that, the short fragments are connected with sequencing adapters. And, after the agarose gel electrophoresis, the suitable fragment are selected for the PCR amplification as templates. At last, the library could be sequenced using Illumina HiSeq™ 2500.

(B) Workflow of the data assembly. Transcriptome de novo assembly is carried out with short reads assembling program-Trinity. Trinity firstly combines reads with certain length of overlap to form longer fragments without N, which are called contigs. Then, these contigs will be taken into further process of sequence cluster with sequence clustering software to form longer sequences without N, Such sequences are defined as Unigenes. When multiple samples from a same species are sequenced, Unigenes from each sample's assembly can be taken into further process of sequence splicing and redundancy removing with sequence clustering software to acquire non-redundant Unigenes as long as possible.
