# Supplementary material for: Transcriptome profiling of litchi leaves in response to low temperature reveals candidate regulatory genes and key metabolic events during floral induction
Source: BMC Genomics. 2017 May 10;18:363. doi: 10.1186/s12864-017-3747-x (PMC5424310; doi:10.1186/s12864-017-3747-x)
Supplement: Supplementary file 3 — Primers of candidate and reference genes used for RT-qPCR. (PDF 90 kb) [file 12864_2017_3747_MOESM3_ESM.pdf]

Additional file 3. Primers of candidate and reference genes used for RT-qPCR

Gene	Forward primer (5' to 3')	Reverse primer (5' to 3')
LcHK	TGATAATAGGAACAGGCACT	TAGGAGAAGCAGGCACAA
LcPFK	AGTTGTTAAGCAGTATTTCGTG	AAACCAGGGCAAAGACCA
LcSPS	CGCTGCTGCTCTTCTATCTG	TGCTCCTCAATCTCCTGTCTAG
LcSS	GGTTGGCACAATAGAAGGT	ATGGCAATGAATGATGTCT
LcSUS	CCTGGTGGTTCTTACGATTACG	CTGCTTCCTGGCTTCATCTTC
LcGBSS	AGCCGTTGCCCAATTTATCAG	TTGCTCAAGCTGTTTCTCCATC
LcbHLH130-1	GTCAAGACTCTCACGGATACG	TCAACTCAATAACCGACCTCTC
LcbHLH130-2	ATGAAGCAAGAAGAGGCAGAG	GCACCAGATTCTCCATTGTTAC
LcGATA	GCAGCAGAAGCAGCAACC	TCACTGTCAGCCAACCAATAAG
LcMYB	GTCAACTCGGTCGCTATCG	GCAGGCACTAATCTCCCATC
LcTCP	ACCTACTAACCAGCCTCACA	TGAAGCCGAGACTGAAGAC
LcbHLH61	AAGAAGCGAAGCAAGTCA	CAAATGTCATCCGTCCAA
LcFT	CAAGACTGAGGGAGTACTTG	GGAGATCCAAGGTTGTAAAGC
LcFLC	TCCTCTTCAAAGCGACTCCG	AGAAAGCCAAGCCACCGTTA
LcFRI	TCGCCAATCTCAATAAACA	CTCTTCCCAACAGCGTAAT
LcVIN3	GTTTGCCAATCACTCCAT	ACAGTCCTCATCCCGTCT
LcCO	GCTTCCTGGCTGTTACCGA	CCATCTGGCACCACTCCTAC
LcSOC1	TCTCGGTTCTTTGTGATGC	TCTCCTAATAGTCTCCGTTTT
LcAP1	TTAGGGCAGCCACCTCAGTC	GTGCAGCAACCTCTTTACATCA
LcVRN1	TTATCTCCTCCACCATCCA	CCAACCGTCATAGAACCAG
LcActin	GTGGTTCTACTATGTTCCCTG	CTCGTCGTACTCATCCTTTG
