# Supplementary material for: Transcriptome profiling of litchi leaves in response to low temperature reveals candidate regulatory genes and key metabolic events during floral induction
Source: BMC Genomics. 2017 May 10;18:363. doi: 10.1186/s12864-017-3747-x (PMC5424310; doi:10.1186/s12864-017-3747-x)
Supplement: Supplementary file 4 — Summary statistics of the sequence assembly. (PDF 31 kb) [file 12864_2017_3747_MOESM4_ESM.pdf]

Additional file 4. Summary statistics of the sequence assembly

Category	Number
Total clean reads	103,924,174
Total clean nucleotides (nt)	10,392,417,400
Q20 percentage	99.14%
N percentage	0.00%
GC percentage	45.7%
Total number of contigs	97,449
Average contig length (bp)	941
Total number of unigenes	83,107
Average unigene length (bp)	1,552
