# Supplementary material for: Transcriptome profiling of litchi leaves in response to low temperature reveals candidate regulatory genes and key metabolic events during floral induction
Source: BMC Genomics. 2017 May 10;18:363. doi: 10.1186/s12864-017-3747-x (PMC5424310; doi:10.1186/s12864-017-3747-x)
Supplement: Supplementary file 5 — Length distribution and number of Contigs (A) and Unigenes (B) assembly. The horizontal coordinates are lengths and the vertical coordinates are numbers of Contigs and Unigenes. (PDF 19 kb) [file 12864_2017_3747_MOESM5_ESM.pdf]

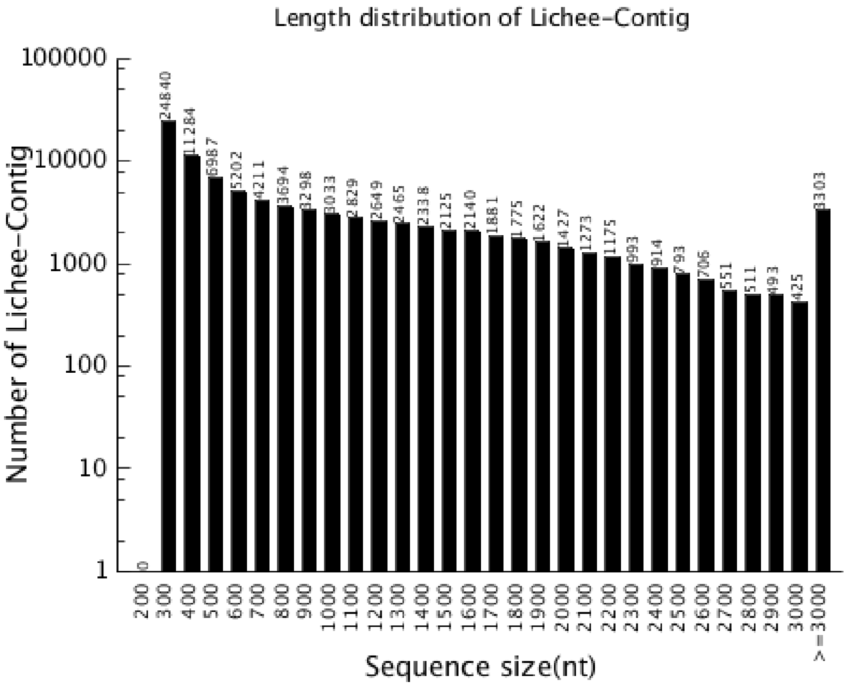

A

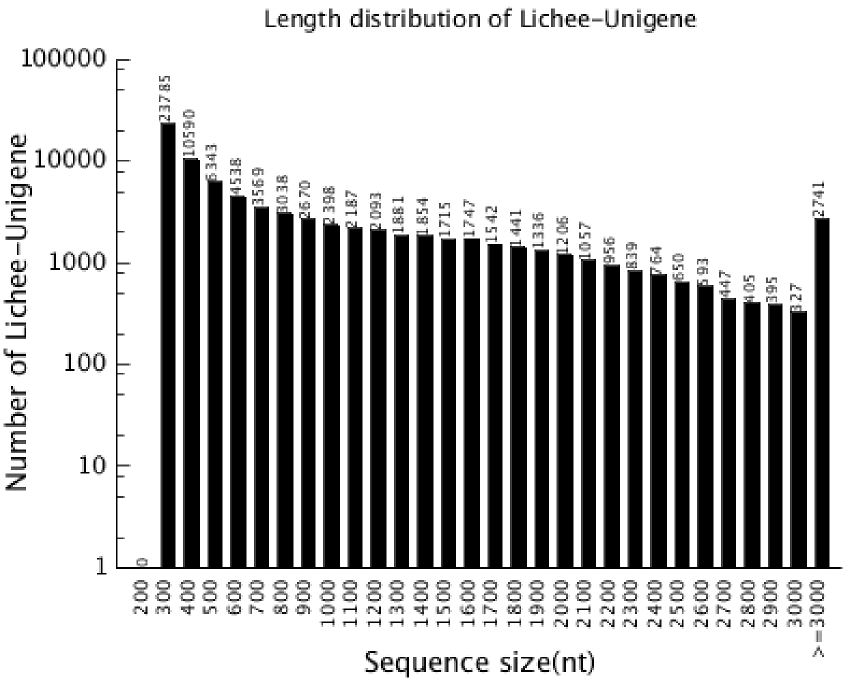

B

Additional file 5. Length distribution and number of Contigs (A) and Unigenes (B) assembly.

The horizontal coordinates are lengths and the vertical coordinates are numbers of Contigs and Unigenes.
