# Supplementary material for: Transcriptome profiling of litchi leaves in response to low temperature reveals candidate regulatory genes and key metabolic events during floral induction
Source: BMC Genomics. 2017 May 10;18:363. doi: 10.1186/s12864-017-3747-x (PMC5424310; doi:10.1186/s12864-017-3747-x)
Supplement: Supplementary file 6 — Histogram of GO classifications for litchi leaf transcriptome. The unigenes were assigned to three main categories: biological process, cellular component, and molecular function. The left and right-hand y-axes indicate the percentage and number of annotated unigenes respectively. (PDF 224 kb) [file 12864_2017_3747_MOESM6_ESM.pdf]

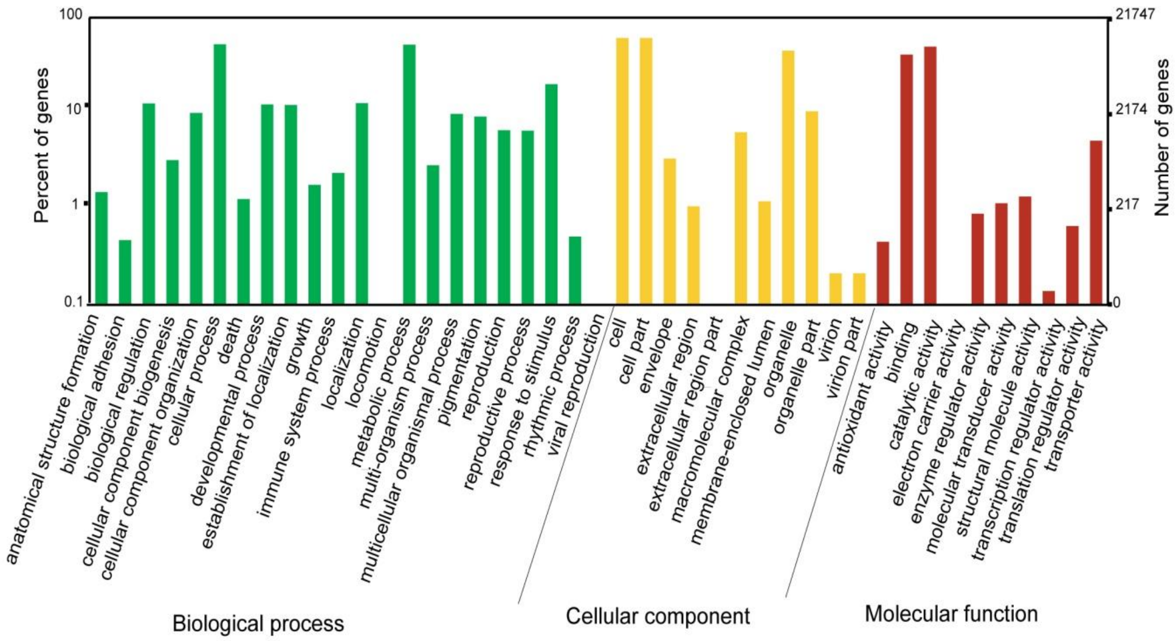

Additional file 6. Histogram of GO classifications for litchi leaf transcriptome.

The unigenes were assigned to three main categories: biological process, cellular component, and molecular function. The left and right-hand y-axes indicate the percentage and number of annotated unigenes respectively.
