# Supplementary material for: Transcriptome profiling of litchi leaves in response to low temperature reveals candidate regulatory genes and key metabolic events during floral induction
Source: BMC Genomics. 2017 May 10;18:363. doi: 10.1186/s12864-017-3747-x (PMC5424310; doi:10.1186/s12864-017-3747-x)
Supplement: Supplementary file 7 — COG functional classifications of the litchi leaf transcriptome. COG is a database where orthologous gene products are classified. Every protein in COG is assumed to evolve from an ancestor protein, and the whole database is built on coding proteins with complete genome as well as system evolution relationships of bacteria, algae and eukaryotic creatures. Unigenes are aligned to COG database to predict and classify possible functions of Unigenes. A total of 18,497 unigenes were classified into 25 COG categories. (PDF 54 kb) [file 12864_2017_3747_MOESM7_ESM.pdf]

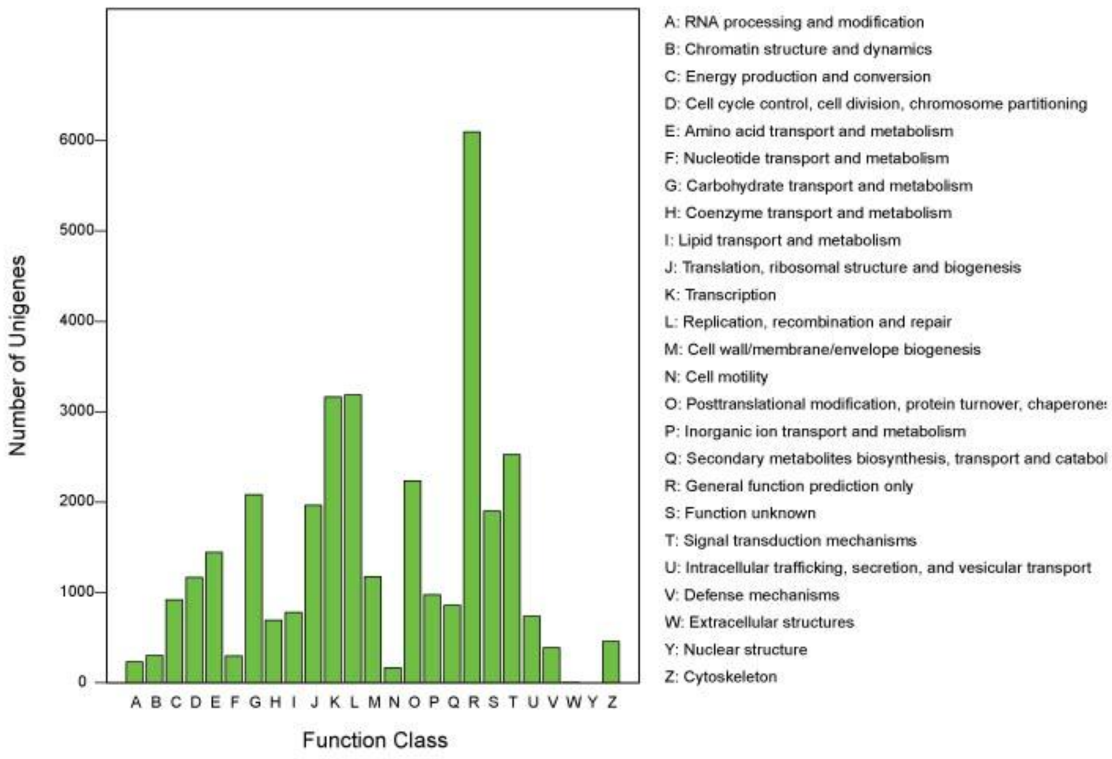

Additional file 7. COG functional classifications of the litchi leaf transcriptome.

COG is a database where orthologous gene products are classified. Every protein in COG is assumed to evolve from an ancestor protein, and the whole database is built on coding proteins with complete genome as well as system evolution relationships of bacteria, algae and eukaryotic creatures. Unigenes are aligned to COG database to predict and classify possible functions of Unigenes. A total of 18,497 unigenes were classified into 25 COG categories.
