# Supplementary material for: Transcriptome profiling of litchi leaves in response to low temperature reveals candidate regulatory genes and key metabolic events during floral induction
Source: BMC Genomics. 2017 May 10;18:363. doi: 10.1186/s12864-017-3747-x (PMC5424310; doi:10.1186/s12864-017-3747-x)
Supplement: Supplementary file 8 — Pathway assignment based on KEGG. (PDF 24 kb) [file 12864_2017_3747_MOESM8_ESM.pdf]

Additional file 8. Pathway assignment based on KEGG

No.	Pathway	Number of annotation	Pathway ID
1	RNA transport	434 (3.25%)	ko03013
2	Ribosome	348 (2.61%)	ko03010
3	Starch and sucrose metabolism	344 (2.58%)	ko00500
4	Purine metabolism	323 (2.42%)	ko00230
5	Plant-pathogen interaction	323 (2.42%)	ko04626
6	Protein processing in endoplasmic reticulum	309 (2.31%)	ko04141
7	Spliceosome	303 (2.27%)	ko03040
8	Plant hormone signal transduction	303 (2.27%)	ko04075
9	Glycolysis / Gluconeogenesis	302 (2.26%)	ko00010
10	Cell cycle	294 (2.2%)	ko04110
11	Ubiquitin mediated proteolysis	289 (2.16%)	ko04120
12	Ribosome biogenesis in eukaryotes	279 (2.09%)	ko03008
13	Cell cycle - yeast	268 (2.01%)	ko04111
14	Methane metabolism	242 (1.81%)	ko00680
15	Oxidative phosphorylation	241 (1.8%)	ko00190
16	Pyrimidine metabolism	227 (1.7%)	ko00240
17	Meiosis - yeast	222 (1.66%)	ko04113
18	Oocyte meiosis	222 (1.66%)	ko04114
19	RNA degradation	221 (1.65%)	ko03018
20	mRNA surveillance pathway	210 (1.57%)	ko03015
21	Peroxisome	208 (1.56%)	ko04146
22	Amino sugar and nucleotide sugar metabolism	183 (1.37%)	ko00520
23	Valine, leucine and isoleucine degradation	182 (1.36%)	ko00280
24	Arginine and proline metabolism	177 (1.33%)	ko00330
25	Phenylpropanoid biosynthesis	174 (1.3%)	ko00940
26	Lysosome	172 (1.29%)	ko04142

27	Fructose and mannose metabolism	168 (1.26%)	ko00051
28	Galactose metabolism	167 (1.25%)	ko00052
29	Cysteine and methionine metabolism	164 (1.23%)	ko00270
30	Huntington's disease	163 (1.22%)	ko05016
31	Pyruvate metabolism	161 (1.21%)	ko00620
32	Glycerophospholipid metabolism	159 (1.19%)	ko00564
33	Inositol phosphate metabolism	159 (1.19%)	ko00562
34	Endocytosis	158 (1.18%)	ko04144
35	Alzheimer's disease	155 (1.16%)	ko05010
36	Phagosome	151 (1.13%)	ko04145
37	Carbon fixation in photosynthetic organisms	147 (1.1%)	ko00710
38	Aminoacyl-tRNA biosynthesis	147 (1.1%)	ko00970
39	Pentose phosphate pathway	146 (1.09%)	ko00030
40	Glutathione metabolism	141 (1.06%)	ko00480
