# Supplementary material for: Transcriptome profiling of litchi leaves in response to low temperature reveals candidate regulatory genes and key metabolic events during floral induction
Source: BMC Genomics. 2017 May 10;18:363. doi: 10.1186/s12864-017-3747-x (PMC5424310; doi:10.1186/s12864-017-3747-x)
Supplement: Supplementary file 9 — Sequencing Assessment. (A) Sequencing saturation analysis result; (B) Distribution statistics of reads mapped to reference gene; (C) Gene coverage statistics. (PDF 176 kb) [file 12864_2017_3747_MOESM9_ESM.pdf]

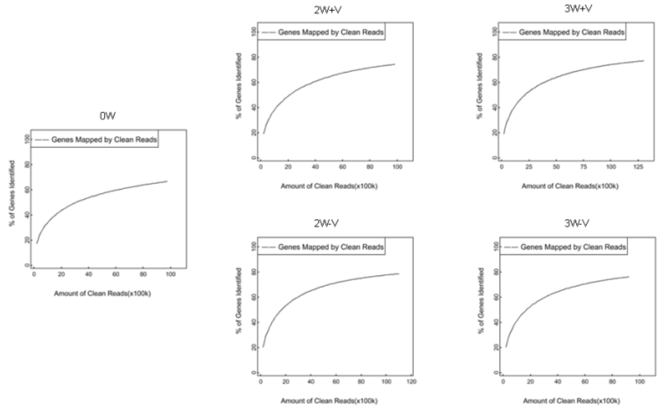

A

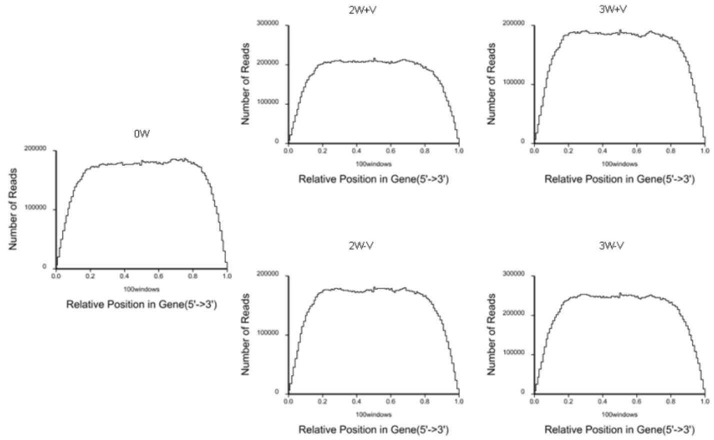

B

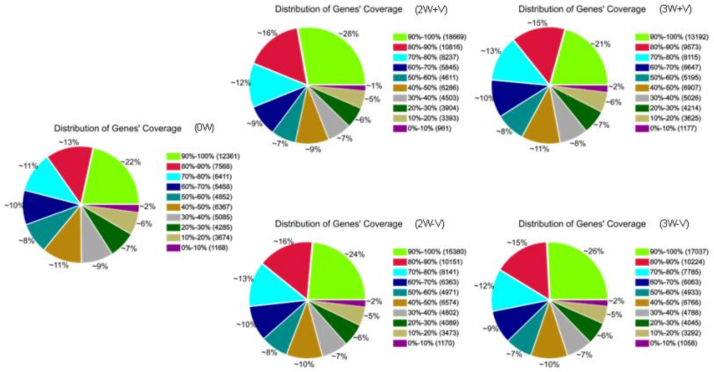

C

Additional file 9. Sequencing Assessment.

(A) Sequencing saturation analysis result; (B) Distribution statistics of reads mapped to reference gene; (C) Gene coverage statistics.
