# Supplementary material for: Transcriptome profiling of litchi leaves in response to low temperature reveals candidate regulatory genes and key metabolic events during floral induction
Source: BMC Genomics. 2017 May 10;18:363. doi: 10.1186/s12864-017-3747-x (PMC5424310; doi:10.1186/s12864-017-3747-x)
Supplement: Supplementary file 10 — All STEM profiles by significance. Profiles ordered based on the p-value significance of number of genes were assigned versus expected. The ID and gene numbers of profiles were showed on the top of frames. P-values were represented with the number inside. Colored figures denoted the p-value (p < 0.05) significance, and the similar trends were set with the same color. (PDF 104 kb) [file 12864_2017_3747_MOESM10_ESM.pdf]

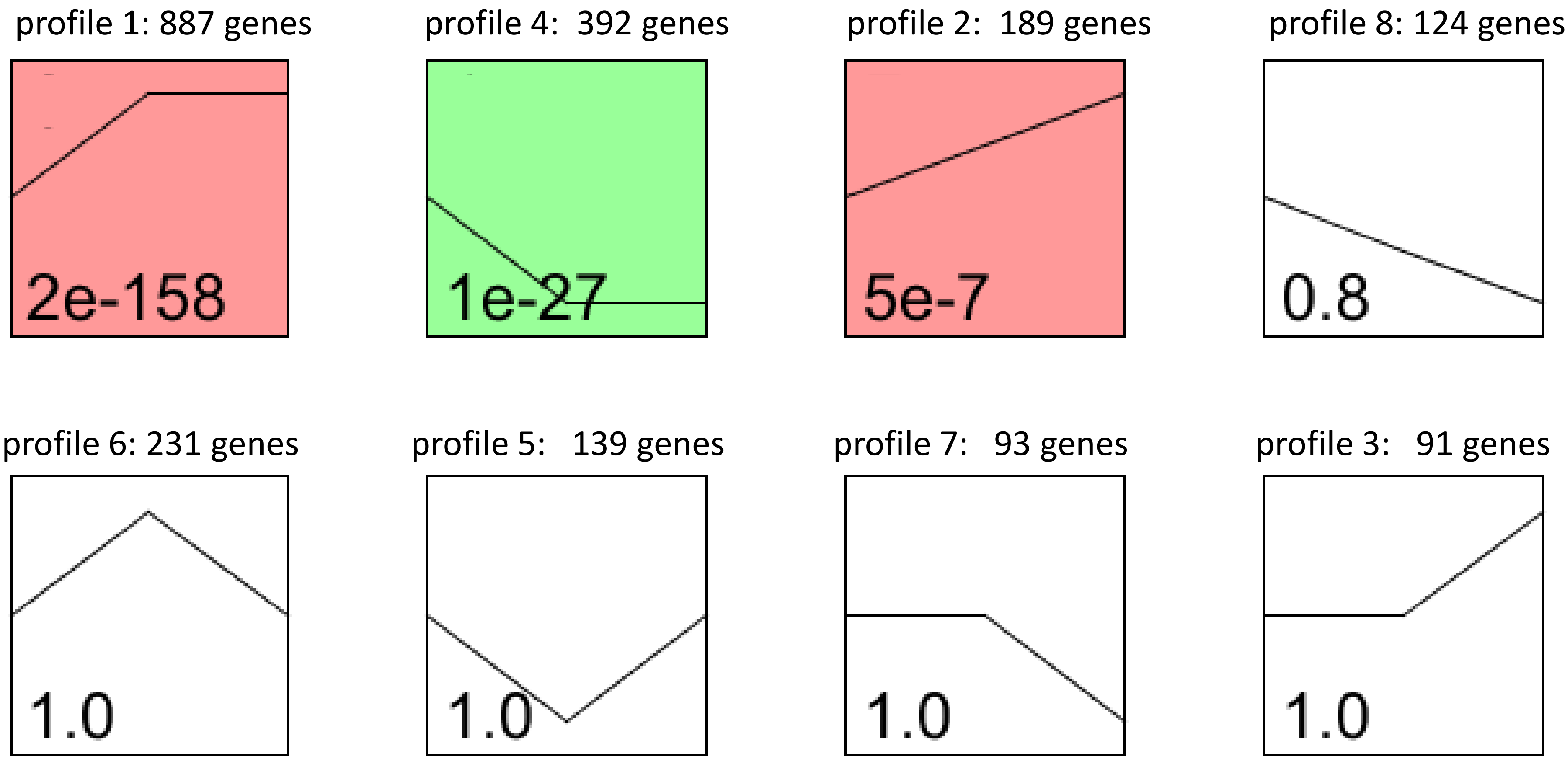

Additional file 10. All STEM profiles by significance.

Profiles ordered based on the p-value significance of number of genes assigened versus expected. The ID and gene numbers of profiles were showed on the top of frames. P-values were represented with the number inside. Colored figures denoted the p-value ($p<0.05$) significance, and the similar trends were setted with the same color.
