# Supplementary material for: Transcriptome profiling of litchi leaves in response to low temperature reveals candidate regulatory genes and key metabolic events during floral induction
Source: BMC Genomics. 2017 May 10;18:363. doi: 10.1186/s12864-017-3747-x (PMC5424310; doi:10.1186/s12864-017-3747-x)
Supplement: Supplementary file 11 — Hierarchical clustering analysis and category distribution of DGEs during floral induction. 276 modulated DEGs were obtained from the gene expression profiles of only significantly differentially expressed under low temperature treatment, but no significant change in control treatment. The RPKM for significantly DEGs was used, the up-regulated and down-regulated genes are indicated in red and green respectively. The grids with different ranks from left to right showed 0W, 2W + V, 3W + V, 2W-V and 3W-V. (PDF 247 kb) [file 12864_2017_3747_MOESM11_ESM.pdf]

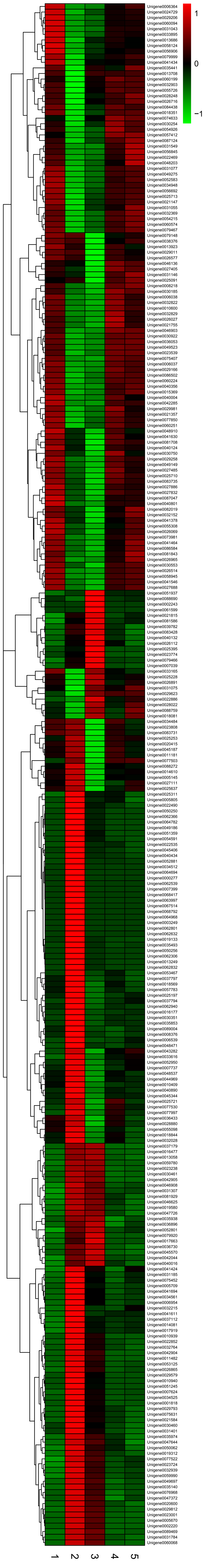

1
0
−1
1
2
3
4
5

Additional file 11. Hierarchical clustering analysis and category distribution of DGEs during floral induction. 276 modulated DEGs were obtained from the gene expression profiles of only significantly differentially expressed under low temperature treatment, but no significant change in control treatment. The $\log_2$ Ratio for significantly DEGs was used, the up-regulated and down-regulated genes are indicated in red and green respectively. The grids with different ranks from left to right showed 0W, 2W+V, 3W+V, 2W-V and 3W-V.
