# Supplementary material for: Transcriptome profiling of litchi leaves in response to low temperature reveals candidate regulatory genes and key metabolic events during floral induction
Source: BMC Genomics. 2017 May 10;18:363. doi: 10.1186/s12864-017-3747-x (PMC5424310; doi:10.1186/s12864-017-3747-x)
Supplement: Supplementary file 12 — Simplified diagram showing the four major genetic pathways regulating the floral transition in Arabidopsis (Boss et al. 2004; Putterill et al. 2004). Arrows indicate activation and straight lines ending with a perpendicular line indicate repression. The numbers below each gene represent the members of the corresponding genes found from RNA-Seq data for litchi. (PDF 129 kb) [file 12864_2017_3747_MOESM12_ESM.pdf]

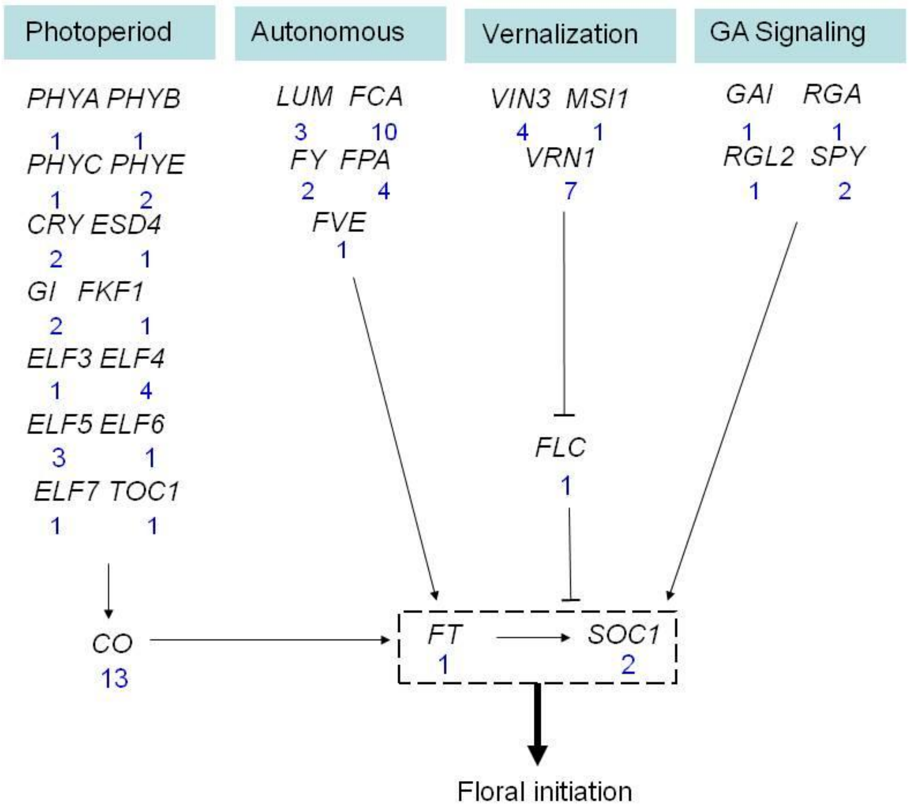

Additional file 12. Simplified diagram showing the four major genetic pathways regulating the floral transition in Arabidopsis (Boss et al. 2004; Putterill et al. 2004).

Arrows indicate activation and straight lines ending with a perpendicular line indicate repression. All the corresponding homologs of litchi involved in these flowering pathways are listed in Additional file 10. The numbers below each gene represent the members of the corresponding genes found from RNA-Seq data for litchi.
