# Supplementary material for: Transcriptome profiling of litchi leaves in response to low temperature reveals candidate regulatory genes and key metabolic events during floral induction
Source: BMC Genomics. 2017 May 10;18:363. doi: 10.1186/s12864-017-3747-x (PMC5424310; doi:10.1186/s12864-017-3747-x)
Supplement: Supplementary file 13 — Putative orthologs of litchi genes involved in flowering induction. (PDF 139 kb) [file 12864_2017_3747_MOESM13_ESM.pdf]

Additional file 13. Putative orthologs of litchi genes involved in flowering induction

GeneID	0W (RPKM)	2W+V (RPKM)	3W+V (RPKM)	2W-V (RPKM)	3W-V (RPKM)	Annotation
**Autonomous pathway**						
Unigene0054377	8.7	10.0	9.1	9.3	8.2	LUM [Vitis vinifera]
Unigene0054378	1.9	2.9	2.9	3.9	2.6	LUM [Glycine max]
Unigene0054379	0.3	0.7	0.5	1.5	0.7	LUM [Vitis vinifera]
Unigene0086608	44.6	43.3	43.0	42.5	50.7	FCA [Vitis vinifera]
Unigene0025131	35.2	36.4	39.4	34.8	28.9	FCA [Vitis vinifera]
Unigene0043090	0.6	0.4	0.3	0.1	0.2	FCA [Glycine max]
Unigene0043094	0.6	2.0	1.3	0.5	0.7	FCA [Fragaria vesca]
Unigene0043097	0.3	2.2	0.6	0.9	0.9	FCA [Fragaria vesca]
Unigene0043096	0.1	0.2	0.3	0.2	0.1	FCA [Glycine max]
Unigene0043095	0.1	-	0.2	0.1	0.1	FCA [Glycine max]
Unigene0025132	0.7	1.3	2.2	0.3	2.3	FCA [Vitis vinifera]
Unigene0043093	-	0.2	0.1	-	0.1	FCA [Fragaria vesca]
Unigene0043089	-	0.3	-	-	-	FCA [Fragaria vesca]
Unigene0061002	0.4	2.5	3.8	2.0	2.4	FY [Medicago truncatula]
Unigene0060136	1.2	4.2	3.4	3.5	3.7	FY [Vitis vinifera]
Unigene0081624	10.9	9.1	10.9	8.7	12.6	FPA [Cicer arietinum]
Unigene0088521	0.8	0.4	0.5	0.5	0.6	FPA [Glycine max]
Unigene0088522	0.7	0.6	0.8	1.1	0.7	FPA [Glycine max]
Unigene0052266	2.3	7.0	6.9	5.3	5.2	FPA [Glycine max]
Unigene0040819	57.1	80.8	89.0	86.6	82.3	FVE [Litchi chinensis]
**Vernalization pathway**						
Unigene0035533	34.7	70.7	60.8	63.0	63.6	VRN1 [Vitis vinifera]
Unigene0037957	0.2	0.6	0.7	0.5	0.5	VRN1 [Vitis vinifera]
Unigene0037958	-	0.5	0.6	1.4	0.8	VRN1 [Vitis vinifera]
Unigene0075163	1.1	4.7	2.5	4.2	4.4	VRN1 [Fragaria vesca]
Unigene0086852	1.9	3.7	4.4	4.8	3.6	VRN1 [Cucumis sativus]
Unigene0021971	5.0	25.1	22.9	18.1	18.3	VRN1 [Cicer arietinum]

Unigene0024099	0.2	1.1	1.0	2.3	1.1	VRN1 [Cicer arietinum]
Unigene0067329	-	0.7	0.4	0.4	0.6	VIN3 [Vitis vinifera]
Unigene0089124	0.7	1.0	1.0	1.1	1.2	VIN3 [Vitis vinifera]
Unigene0089124	0.7	1.0	1.1	1.0	1.2	VIN3 [Vitis vinifera]
Unigene0045531	22.0	16.9	17.8	20.9	19.0	VIN3 [Glycine max]
Unigene0034232	1.1	0.1	0.4	0.5	0.2	MSI1 [Cucumis sativus]
Unigene0037830	53.6	46.7	43.4	45.0	39.7	FLC [Citrus trifoliata]
**Photoperiod pathway**						
Unigene0038504	0.1	0.2	0.4	0.3	0.4	TOC1 [Castanea sativa]
Unigene0051713	2.7	9.8	9.2	8.5	9.7	PHYE [Theobroma cacao]
Unigene0024396	2.7	5.4	6.7	6.4	5.5	PHYE [Theobroma cacao]
Unigene0082114	7.1	17.1	15.8	16.7	14.4	PHYC [Theobroma cacao]
Unigene0053719	4.0	16.0	14.1	15.4	12.6	PHYB [Theobroma cacao]
Unigene0086802	2.0	11.3	8.5	9.9	7.9	PHYA [Theobroma cacao]
Unigene0077682	245.2	438.9	658.5	520.3	592.9	GI [Theobroma cacao]
Unigene0046169	134.0	246.7	361.4	202.2	217.1	GI [Fragaria vesca]
Unigene0048122	10.5	103.6	104.0	86.5	81.2	FKF1 [Glycine max]
Unigene0056472	2.3	9.6	6.9	8.4	7.4	ELF7 [Vitis vinifera]
Unigene0057587	6.5	10.5	13.5	11.6	16.6	ELF6 [Vitis vinifera]
Unigene0056473	4.4	5.6	5.7	6.1	5.9	ELF5 [Vitis vinifera]
Unigene0041580	56.4	39.5	43.0	48.6	51.4	ELF5 [Citrus trifoliata]
Unigene0041579	58.3	42.8	49.8	46.5	48.0	ELF5 [Citrus trifoliata]
Unigene0074891	8.5	14.9	15.9	15.6	22.6	ELF4 [Vitis vinifera]
Unigene0037820	44.0	42.8	105.7	63.6	73.0	ELF4 [Manihot esculenta]
Unigene0031962	36.2	15.5	23.2	21.6	18.9	ELF4 [Ipomoea nil]
Unigene0053929	16.1	20.2	25.5	15.9	21.5	ELF4 [Citrus sinensis]
Unigene0057588	30.0	42.0	51.7	47.8	65.2	ELF3 [Vitis vinifera]
Unigene0083829	0.2	0.7	0.8	4.7	4.8	CRY [Populus tremula]
Unigene0056595	0.2	1.4	1.5	1.0	0.7	CRY [Populus tremula]
Unigene0035119	18.4	396.2	247.3	314.5	240.4	CO4 [Theobroma cacao]
Unigene0026093	8.3	70.2	24.6	75.5	27.5	CO2 [Theobroma cacao]

Unigene0048016	2.3	21.7	8.8	20.2	10.2	CO2 [Theobroma cacao]
Unigene0048017	0.5	4.2	0.8	2.0	1.2	CO2 [Theobroma cacao]
Unigene0028510	6.3	228.1	205.1	257.5	224.6	CO16 [Vitis vinifera]
Unigene0028510	6.3	228.1	205.1	257.5	224.6	CO16 [Vitis vinifera]
Unigene0022621	0.5	29.1	18.9	17.6	14.4	CO16 [Vitis vinifera]
Unigene0050297	34.4	31.6	42.4	28.3	46.3	CO14 [Vitis vinifera]
Unigene0033519	5.6	2.8	1.8	0.6	2.6	CO14 [Vitis vinifera]
Unigene0075371	20.9	19.9	23.3	22.8	26.0	CO13 [Vitis vinifera]
Unigene0077823	15.5	4.2	9.3	6.1	16.6	CO [Medicago truncatula]
Unigene0084415	16.8	90.5	114.8	93.8	133.7	CO [Medicago truncatula]
Unigene0039395	14.9	289.2	164.9	224.0	105.9	CO [Litchi chinensis]
**Gibberellin pathway**						
Unigene0033523	53.9	70.3	51.0	57.1	50.6	GAI [Juglans regia]
Unigene0040525	14.8	23.9	36.3	24.5	29.0	RGA [Gossypium hirsutum]
Unigene0010096	0.4	2.8	0.4	-	0.9	RGL2 [Vitis vinifera]
Unigene0049693	3.3	11.2	9.2	8.9	9.8	SPY [Malus domestica]
Unigene0049691	1.8	6.2	3.7	6.0	3.0	SPY [Malus domestica]
**Floral pathway integrator**						
Unigene0033852	1.7	3.6	4.3	2.6	3.2	SOC1 [Citrus sinensis]
Unigene0075092	0.8	2.3	1.1	0.9	1.6	SOC1 [Citrus sinensis]
Unigene0025396	-	0.6	11.4	0.4	1.1	FT [Litchi chinensis]
