# Supplementary material for: Transcriptome profiling of litchi leaves in response to low temperature reveals candidate regulatory genes and key metabolic events during floral induction
Source: BMC Genomics. 2017 May 10;18:363. doi: 10.1186/s12864-017-3747-x (PMC5424310; doi:10.1186/s12864-017-3747-x)
Supplement: Supplementary file 14 — RT-qPCR verification of differential expression. The figure shows transcript levels of 14 genes, of which 6 probably associated with transcription factors (A) and 8 with flowering-related genes (B) under 15 °C (black) and 25 °C (white) treatment by comparing of RT-qPCR data (bar) with RNA-seq data (line). The relative RT-qPCR expression level is shown on the y-axis to the left, bars represent the standard error (n = 3), and the normalized expression level (RPKM) of RNA-seq is indicated on the y-axis to the right. Actin was used as the internal control. (C) Coefficient analysis between gene expression ratios obtained from RNA-seq data and RT-qPCR. The RT-qPCR log2 expression ratios (x-axis) were plotted against RNA-seq data ratios (y-axis). (PDF 160 kb) [file 12864_2017_3747_MOESM14_ESM.pdf]

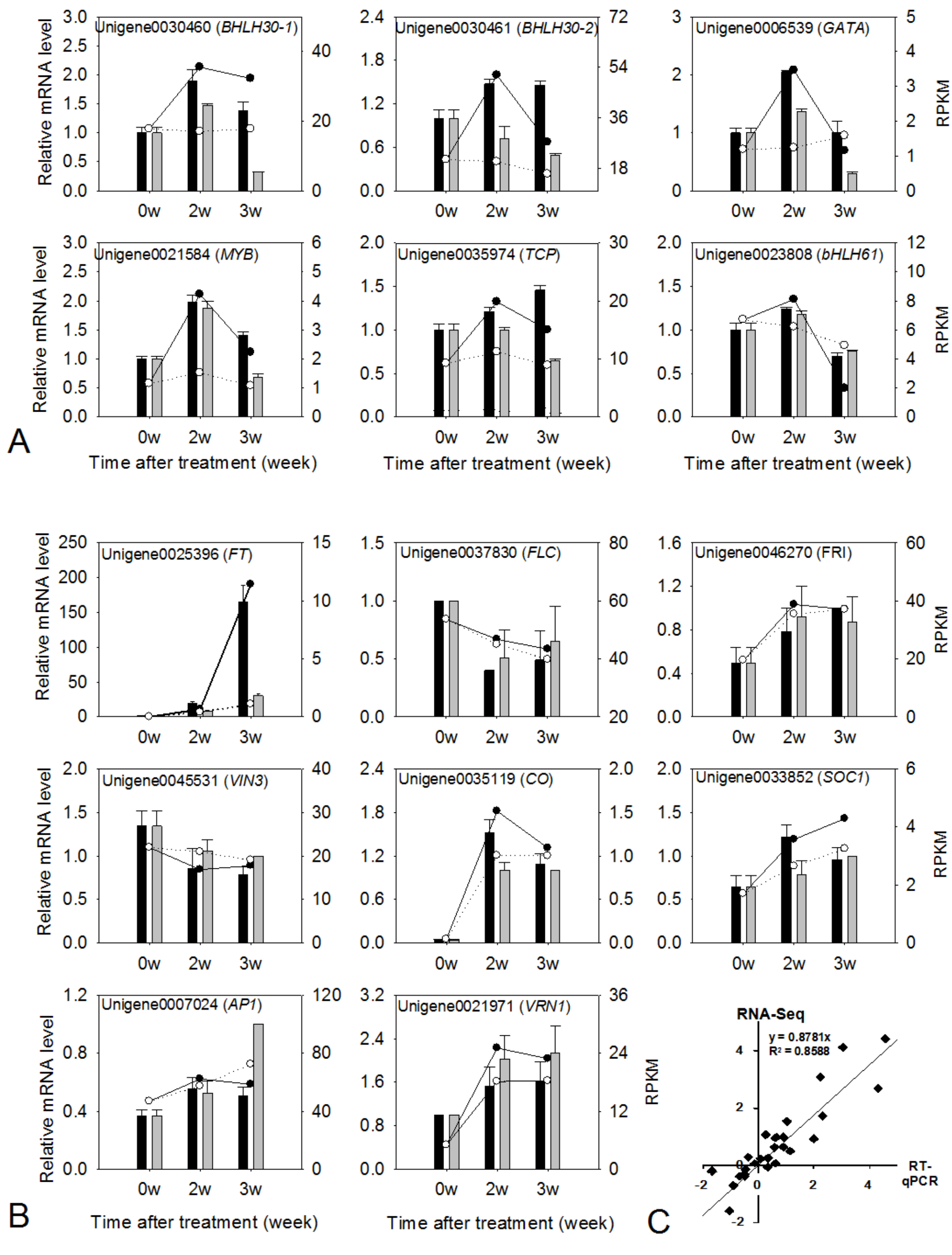

Additional file 14. RT-qPCR verification of differential expression.

The figure shows transcript levels of 14 genes, of which 6 probably associated with transcription factors (A) and 8 with flowering-related genes (B) under 15℃ (black) and 25℃ (white) treatment by comparing of RT-qPCR data (bar) with RNA-seq data (line). The relative RT-qPCR expression level is shown on the y-axis to the left, bars represent the standard error (n=3), and the normalized expression level (RPKM) of RNA-seq is indicated on the y-axis to the right. Actin was used as the internal control. (C) Coefficient analysis between gene expression ratios obtained from RNA-seq data and RT-qPCR. The RT-qPCR log_2 expression ratios (x-axis) were plotted against RNA-seq data ratios (y-axis).
