# Supplementary material for: Transcriptome profiling of litchi leaves in response to low temperature reveals candidate regulatory genes and key metabolic events during floral induction
Source: BMC Genomics. 2017 May 10;18:363. doi: 10.1186/s12864-017-3747-x (PMC5424310; doi:10.1186/s12864-017-3747-x)
Supplement: Supplementary file 15 — Analysis of FT homologous transcripts in litchi. (A) Alignment of the FT homologues proteins. (B) Phylogenetic relationship between Unigene0025396 and other FT proteins from higher plant species. The accession numbers of the sequences used for the alignment are: LcFT1 [Litchi chinensis, AEU08960.1], LcFT2 [Litchi chinensis, AEU08961.1], DlFT1 [Dimocarpus longan, AEZ63949.1], DlFT2 [Dimocarpus longan, AEZ63950.1], MdFT1 [Malus x domestica, BAD08340.1], VvFT1 [Vitis vinifera, ABL98120.1], AtFT [Arabidopsis thaliana, BAA77838], CuFT [Citrus unshiu, BAF96644.1], PtFT [Populus tomentosa, AFU08239.1] and GmFT [Glycine max, BAJ33489.1]. (PDF 231 kb) [file 12864_2017_3747_MOESM15_ESM.pdf]

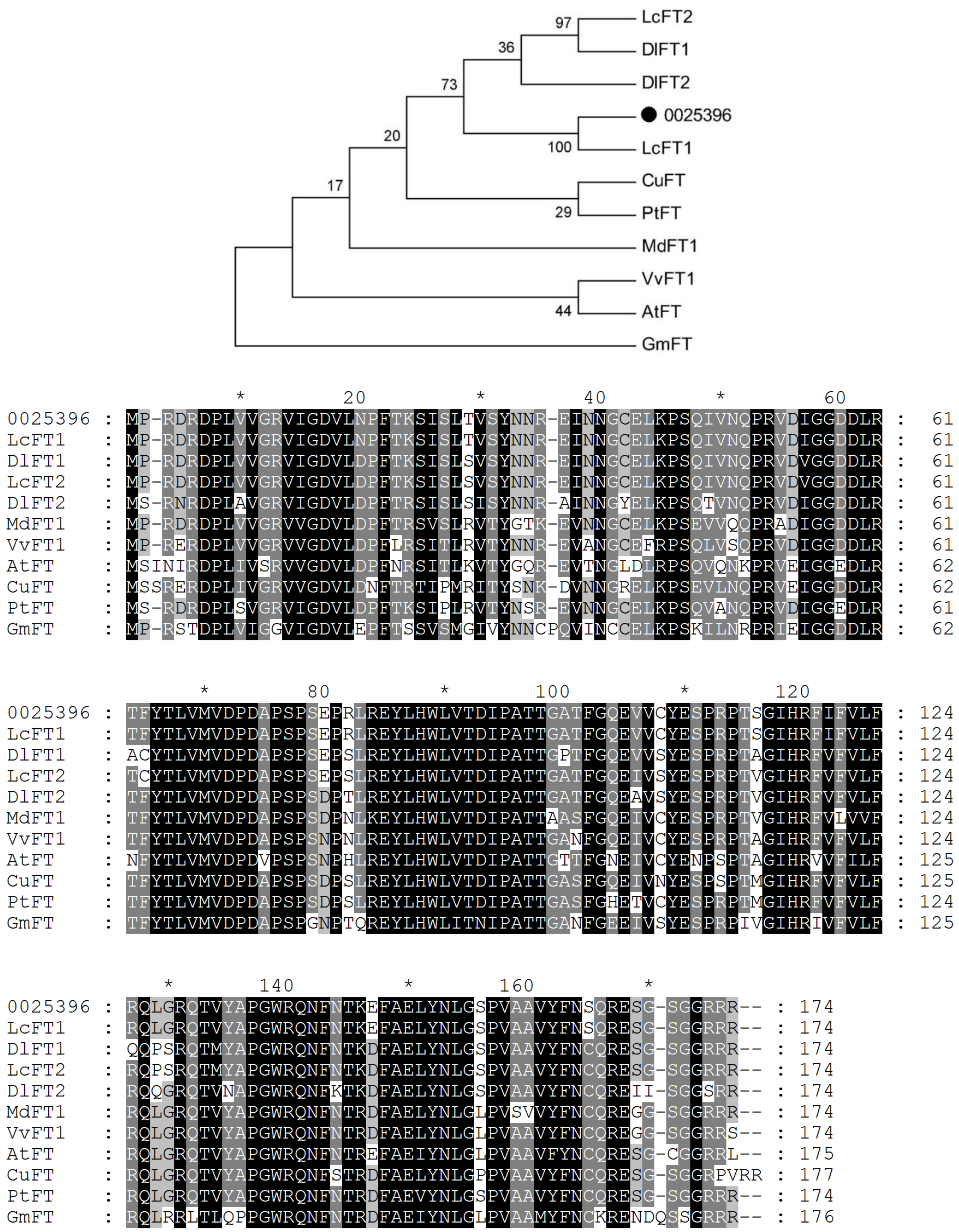

Additional file 15. Analysis of FT homologous transcripts in litchi.

(A) Alignment of the FT homologues proteins. (B) Phylogenetic relationship between Unigene0025396 and other FT proteins from higher plant species. The accession numbers of the sequences used for the alignment are: LcFT1 [*Litchi chinensis*, AEU08960.1], LcFT2 [*Litchi chinensis*, AEU08961.1], DlFT1 [*Dimocarpus longan*, AEZ63949.1], DlFT2 [*Dimocarpus longan*, AEZ63950.1], MdFT1 [*Malus* x *domestica*, BAD08340.1], VvFT1 [*Vitis vinifera*, ABL98120.1], AtFT [*Arabidopsis thaliana*, BAA77838], CuFT [*Citrus unshiu*, BAF96644.1], PtFT [*Populus tomentosa*, AFU08239.1] and GmFT [*Glycine max*, BAJ33489.1].
