# Supplementary material for: Transcriptome profiling of litchi leaves in response to low temperature reveals candidate regulatory genes and key metabolic events during floral induction
Source: BMC Genomics. 2017 May 10;18:363. doi: 10.1186/s12864-017-3747-x (PMC5424310; doi:10.1186/s12864-017-3747-x)
Supplement: Supplementary file 16 — The analysis of the LcFT1 promoter elements. (PDF 243 kb) [file 12864_2017_3747_MOESM16_ESM.pdf]

Additional file 16. The analysis of the *LcFT1* promoter elements

Site Name	Organism	Position	Strand	Matrix score.	sequence	function
ACE	Petroselinumcrispum	973	-	9	CTAACGTATT	cis-acting element involved in light responsiveness
ACE	Petroselinumcrispum	1121	+	9	AAAACGTTTA	cis-acting element involved in light responsiveness
ARE	Zea mays	881	+	6	TGGTTT	cis-acting regulatory element essential for the anaerobic induction
AT-rich element	Glycine max	622	+	10	ATAGAAATCAA	binding site of AT-rich DNA binding protein (ATBP-1)
Box 4	Petroselinumcrispum	899	+	6	ATTAAT	part of a conserved DNA module involved in light responsiveness
Box 4	Petroselinumcrispum	915	+	6	ATTAAT	part of a conserved DNA module involved in light responsiveness
Box 4	Petroselinumcrispum	907	+	6	ATTAAT	part of a conserved DNA module involved in light responsiveness
Box 4	Petroselinumcrispum	962	+	6	ATTAAT	part of a conserved DNA module involved in light responsiveness
Box 4	Petroselinumcrispum	903	+	6	ATTAAT	part of a conserved DNA module involved in light responsiveness
Box 4	Petroselinumcrispum	911	+	6	ATTAAT	part of a conserved DNA module involved in light responsiveness
Box I	Pisumsativum	1061	+	7	TTTCAAA	light responsive element
CAAT-box	Arabidopsis thaliana	133	+	5	CCAAT	common cis-acting element in promoter

						and enhancer regions
CAAT-box	Hordeumvulgare	134	+	4	CAAT	common cis-acting element in promoter and enhancer regions
CAAT-box	Hordeumvulgare	142	-	4	CAAT	common cis-acting element in promoter and enhancer regions
CAAT-box	Hordeumvulgare	166	-	4	CAAT	common cis-acting element in promoter and enhancer regions
CAAT-box	Hordeumvulgare	225	+	4	CAAT	common cis-acting element in promoter and enhancer regions
CAAT-box	Hordeumvulgare	233	+	4	CAAT	common cis-acting element in promoter and enhancer regions
CAAT-box	Brassica rapa	255	-	5	CAAAT	common cis-acting element in promoter and enhancer regions
CAAT-box	Brassica rapa	414	-	5	CAAAT	common cis-acting element in promoter and enhancer regions
CAAT-box	Hordeumvulgare	423	+	4	CAAT	common cis-acting element in promoter and enhancer regions
CAAT-box	Brassica rapa	482	-	5	CAAAT	common cis-acting element in promoter and enhancer regions
CAAT-box	Glycine max	548	+	5	CAATT	common cis-acting element in promoter and enhancer regions
CAAT-box	Arabidopsis thaliana	557	+	5	CCAAT	common cis-acting element in promoter and enhancer regions
CAAT-box	Hordeumvulgare	558	+	4	CAAT	common cis-acting element in promoter and enhancer regions
CAAT-box	Brassica rapa	630	+	5	CAAAT	common cis-acting element in promoter and enhancer regions
CAAT-box	Hordeumvulgare	665	-	4	CAAT	common cis-acting element in promoter and enhancer regions
CAAT-box	Brassica rapa	686	-	5	CAAAT	common cis-acting

						element in promoter and enhancer regions
CAAT-box	Arabidopsis thaliana	720	-	5	CCAAT	common cis-acting element in promoter and enhancer regions
CAAT-box	Hordeumvulgare	743	+	4	CAAT	common cis-acting element in promoter and enhancer regions
CAAT-box	Brassica rapa	750	+	5	CAAAT	common cis-acting element in promoter and enhancer regions
CAAT-box	Brassica rapa	925	-	5	CAAAT	common cis-acting element in promoter and enhancer regions
CAAT-box	Hordeumvulgare	940	+	4	CAAT	common cis-acting element in promoter and enhancer regions
CAAT-box	Glycine max	952	-	5	CAATT	common cis-acting element in promoter and enhancer regions
CAAT-box	Hordeumvulgare	953	-	4	CAAT	common cis-acting element in promoter and enhancer regions
CAAT-box	Glycine max	1007	+	5	CAATT	common cis-acting element in promoter and enhancer regions
CAAT-box	Brassica rapa	1030	-	5	CAAAT	common cis-acting element in promoter and enhancer regions
CAAT-box	Glycine max	1037	+	5	CAATT	common cis-acting element in promoter and enhancer regions
CAAT-box	Brassica rapa	1064	+	5	CAAAT	common cis-acting element in promoter and enhancer regions
CAAT-box	Hordeumvulgare	1091	-	4	CAAT	common cis-acting element in promoter and enhancer regions
CAAT-box	Hordeumvulgare	1098	-	4	CAAT	common cis-acting element in promoter and enhancer regions
CAAT-box	Arabidopsis thaliana	1187	+	5	CCAAT	common cis-acting element in promoter and enhancer regions

CAAT-box	Hordeumvulgare	1188	+	4	CAAT	common cis-acting element in promoter and enhancer regions
CAAT-box	Glycine max	1227	+	5	CAATT	common cis-acting element in promoter and enhancer regions
CAAT-box	Brassica rapa	1282	+	5	CAAAT	common cis-acting element in promoter and enhancer regions
CAAT-box	Hordeumvulgare	1407	+	4	CAAT	common cis-acting element in promoter and enhancer regions
CAAT-box	Hordeumvulgare	1418	+	4	CAAT	common cis-acting element in promoter and enhancer regions
CAAT-box	Glycine max	1454	-	5	CAATT	common cis-acting element in promoter and enhancer regions
CAAT-box	Hordeumvulgare	1455	-	4	CAAT	common cis-acting element in promoter and enhancer regions
CAAT-box	Hordeumvulgare	1478	-	4	CAAT	common cis-acting element in promoter and enhancer regions
G-Box	Pisumsativum	931	+	6	CACGTT	cis-acting regulatory element involved in light responsiveness
G-box	Brassica oleracea	280	+	9	TAACACGTAG	cis-acting regulatory element involved in light responsiveness
G-box	Zea mays	931	+	6	CACGTT	cis-acting regulatory element involved in light responsiveness
G-box	Solanumtuberosum	460	+	7	CACATGG	cis-acting regulatory element involved in light responsiveness
GAG-motif	Arabidopsis thaliana	756	-	7	AGAGAGT	part of a light responsive element
GATT-motif	Oryza sativa	249	+	11	CTCCTGATTGGA	part of a light responsive element
GT1-motif	Arabidopsis thaliana	553	-	6	GGTTAA	light responsive element
GT1-motif	Arabidopsis thaliana	1016	+	6	GGTTAA	light responsive element

HSE	Brassica oleracea	1107	-	9	AGAAAATTCG	cis-acting element involved in heat stress responsiveness
LTR	Hordeumvulgare	27	-	6	CCGAAA	cis-acting element involved in low-temperature responsiveness
LTR	Hordeumvulgare	1489	-	6	CCGAAA	cis-acting element involved in low-temperature responsiveness
MBS	Arabidopsis thaliana	69	-	6	CAACTG	MYB binding site involved in drought-inducibility
MBS	Arabidopsis thaliana	1426	-	6	CAACTG	MYB binding site involved in drought-inducibility
MBS	Arabidopsis thaliana	351	+	6	CAACTG	MYB binding site involved in drought-inducibility
Skn-1_motif	Oryza sativa	289	+	5	GTCAT	cis-acting regulatory element required for endosperm expression
Skn-1_motif	Oryza sativa	476	+	5	GTCAT	cis-acting regulatory element required for endosperm expression
Skn-1_motif	Oryza sativa	404	-	5	GTCAT	cis-acting regulatory element required for endosperm expression
TA-rich region	Nicotianatabacum	605	+	20	TATATATATATATATATATA	enhancer
TA-rich region	Nicotianatabacum	609	+	20	TATATATATATATATATATA	enhancer
TA-rich region	Nicotianatabacum	607	+	20	TATATATATATATATATATA	enhancer
TATA-box	Arabidopsis thaliana	47	+	4	TATA	core promoter element around -30 of transcription start
TATA-box	Oryza sativa	49	+	7	TACAAAA	core promoter element around -30 of transcription start

TATA-box	Lycopersiconesculentum	97	+	5	TTTTA	core promoter element around -30 of transcription start
TATA-box	Glycine max	157	+	5	TAATA	core promoter element around -30 of transcription start
TATA-box	Lycopersiconesculentum	162	+	5	TTTTA	core promoter element around -30 of transcription start
TATA-box	Lycopersiconesculentum	176	-	5	TTTTA	core promoter element around -30 of transcription start
TATA-box	Brassica oleracea	227	+	6	ATATAA	core promoter element around -30 of transcription start
TATA-box	Arabidopsis thaliana	228	+	4	TATA	core promoter element around -30 of transcription start
TATA-box	Arabidopsis thaliana	293	-	9	TAAAAATAA	core promoter element around -30 of transcription start
TATA-box	Lycopersiconesculentum	297	+	5	TTTTA	core promoter element around -30 of transcription start
TATA-box	Glycine max	300	+	5	TAATA	core promoter element around -30 of transcription start
TATA-box	Arabidopsis thaliana	357	-	6	TATAAA	core promoter element around -30 of transcription start
TATA-box	Arabidopsis thaliana	358	-	5	TATAA	core promoter element around -30 of transcription start
TATA-box	Arabidopsis thaliana	359	+	4	TATA	core promoter element around -30 of transcription start
TATA-box	Arabidopsis thaliana	368	+	4	TATA	core promoter element around -30 of transcription start
TATA-box	Arabidopsis thaliana	370	+	4	TATA	core promoter element around -30 of transcription start
TATA-box	Lycopersiconesculentum	551	+	5	TTTTA	core promoter element around -30

						of transcription start
TATA-box	Glycine max	573	+	5	TAATA	core promoter element around -30 of transcription start
TATA-box	Arabidopsis thaliana	607	-	9	taTATAAAtc	core promoter element around -30 of transcription start
TATA-box	Brassica napus	608	+	6	ATATAT	core promoter element around -30 of transcription start
TATA-box	Arabidopsis thaliana	609	+	8	TATATATA	core promoter element around -30 of transcription start
TATA-box	Brassica napus	610	+	6	ATATAT	core promoter element around -30 of transcription start
TATA-box	Arabidopsis thaliana	611	+	8	TATATATA	core promoter element around -30 of transcription start
TATA-box	Brassica napus	612	+	6	ATATAT	core promoter element around -30 of transcription start
TATA-box	Arabidopsis thaliana	613	+	8	TATATATA	core promoter element around -30 of transcription start
TATA-box	Brassica napus	614	+	6	ATATAT	core promoter element around -30 of transcription start
TATA-box	Arabidopsis thaliana	615	+	8	TATATATA	core promoter element around -30 of transcription start
TATA-box	Brassica napus	616	+	6	ATATAT	core promoter element around -30 of transcription start
TATA-box	Arabidopsis thaliana	617	+	11	TATAAATATAAA	core promoter element around -30 of transcription start
TATA-box	Brassica napus	618	+	6	ATATAT	core promoter element around -30 of transcription start
TATA-box	Arabidopsis thaliana	619	+	8	TATATATA	core promoter element around -30 of transcription start
TATA-box	Brassica napus	620	+	6	ATATAT	core promoter

						element around -30 of transcription start
TATA-box	Arabidopsis thaliana	621	+	10	taTATAAAtc	core promoter element around -30 of transcription start
TATA-box	Brassica oleracea	622	+	6	ATATAA	core promoter element around -30 of transcription start
TATA-box	Ac	623	+	7	TATAAAT	core promoter element around -30 of transcription start
TATA-box	Lycopersiconesculentum	638	+	5	TTTTA	core promoter element around -30 of transcription start
TATA-box	Glycine max	641	+	5	TAATA	core promoter element around -30 of transcription start
TATA-box	Brassica napus	647	+	6	ATTATA	core promoter element around -30 of transcription start
TATA-box	Arabidopsis thaliana	648	-	5	TATAA	core promoter element around -30 of transcription start
TATA-box	Arabidopsis thaliana	649	+	4	TATA	core promoter element around -30 of transcription start
TATA-box	Pisumsativum	677	-	7	TATATGT	core promoter element around -30 of transcription start
TATA-box	Brassica oleracea	679	+	7	ATATAAT	core promoter element around -30 of transcription start
TATA-box	Arabidopsis thaliana	680	+	4	TATA	core promoter element around -30 of transcription start
TATA-box	Glycine max	682	+	5	TAATA	core promoter element around -30 of transcription start
TATA-box	Lycopersiconesculentum	772	-	5	TTTTA	core promoter element around -30 of transcription start
TATA-box	Glycine max	837	+	5	TAATA	core promoter element around -30 of transcription start

TATA-box	Oryza sativa	842	-	8	TATAAGAA	core promoter element around -30 of transcription start
TATA-box	Arabidopsis thaliana	845	-	7	TATATAA	core promoter element around -30 of transcription start
TATA-box	Arabidopsis thaliana	846	+	4	TATA	core promoter element around -30 of transcription start
TATA-box	Arabidopsis thaliana	848	+	4	TATA	core promoter element around -30 of transcription start
TATA-box	Lycopersiconesculentum	860	+	5	TTTTA	core promoter element around -30 of transcription start
TATA-box	Lycopersiconesculentum	884	+	5	TTTTA	core promoter element around -30 of transcription start
TATA-box	Brassica napus	919	+	6	ATTATA	core promoter element around -30 of transcription start
TATA-box	Arabidopsis thaliana	920	-	5	TATAA	core promoter element around -30 of transcription start
TATA-box	Arabidopsis thaliana	921	+	4	TATA	core promoter element around -30 of transcription start
TATA-box	Lycopersiconesculentum	969	+	5	TTTTA	core promoter element around -30 of transcription start
TATA-box	Glycine max	972	+	5	TAATA	core promoter element around -30 of transcription start
TATA-box	Daucuscarota	1008	-	9	ccTATAAATT	core promoter element around -30 of transcription start
TATA-box	Lycopersiconesculentum	1041	-	5	TTTTA	core promoter element around -30 of transcription start
TATA-box	Brassica napus	1067	+	6	ATATAT	core promoter element around -30 of transcription start
TATA-box	Arabidopsis thaliana	1068	+	4	TATA	core promoter element around -30

						of transcription start
TATA-box	Nicotianatabacum	1072	+	9	tcTATAAAta	core promoter element around -30 of transcription start
TATA-box	Arabidopsis thaliana	1074	+	4	TATA	core promoter element around -30 of transcription start
TATA-box	Lycopersiconesculentum	1120	-	5	TTTTA	core promoter element around -30 of transcription start
TATA-box	Brassica oleracea	1196	+	6	ATATAA	core promoter element around -30 of transcription start
TATA-box	Arabidopsis thaliana	1197	+	4	TATA	core promoter element around -30 of transcription start
TATA-box	Lycopersiconesculentum	1276	-	5	TTTTA	core promoter element around -30 of transcription start
TATA-box	Lycopersiconesculentum	1300	-	5	TTTTA	core promoter element around -30 of transcription start
TATA-box	Arabidopsis thaliana	1371	-	9	tcTATATAtt	core promoter element around -30 of transcription start
TATA-box	Brassica napus	1372	+	6	ATATAT	core promoter element around -30 of transcription start
TATA-box	Arabidopsis thaliana	1373	+	8	TATATATA	core promoter element around -30 of transcription start
TATA-box	Brassica napus	1374	+	6	ATATAT	core promoter element around -30 of transcription start
TATA-box	Arabidopsis thaliana	1375	+	4	TATA	core promoter element around -30 of transcription start
TATA-box	Arabidopsis thaliana	1377	+	4	TATA	core promoter element around -30 of transcription start
TC-rich repeats	Nicotianatabacum	186	+	9	ATTTTCTCCA	cis-acting element involved in defense and stress responsiveness

TCA-element	Brassica oleracea	246	-	9	TCAGAAGAGG	cis-acting element involved in salicylic acid responsiveness
TCA-element	Brassica oleracea	1434	-	9	GAGAAGAATA	cis-acting element involved in salicylic acid responsiveness
TCCC-motif	Spinaciaoleracea	56	-	7	TCTCCCT	part of a light responsive element
circadian	Lycopersiconesculentum	705	+	6	CAANNNNATC	cis-acting regulatory element involved in circadian control
circadian	Lycopersiconesculentum	1064	+	9	CAAAGATATC	cis-acting regulatory element involved in circadian control
